# Mixed methods investigation of the use of telephone triage within UK veterinary practices for horses with abdominal pain: A Participatory action research study

**DOI:** 10.1371/journal.pone.0238874

**Published:** 2020-09-23

**Authors:** Katie L. Lightfoot, John H. Burford, Gary C. W. England, I. Mark Bowen, Sarah L. Freeman

**Affiliations:** School of Veterinary Medicine and Science, University of Nottingham, Loughborough, Leicestershire, United Kingdom; Georgia Southern University, UNITED STATES

## Abstract

**Background:**

Telephone triage is an integral part of modern patient care systems in human medicine, and a key component of veterinary practice care systems. There is currently no published research on telephone triage within the veterinary profession.

**Objective:**

To investigate current approaches to telephone triage of horses with abdominal pain (colic) in veterinary practice and develop new resources to support decision-making.

**Study design:**

Participatory action research using mixed-methods approach.

**Methods:**

An online survey assessed current approaches to telephone triage of horses with colic in UK veterinary practices. Structured group and individual interviews were conducted with four equine client care (reception) teams on their experiences around telephone triage of colic. Evidence-based resources, including an information pack, decision flow chart and recording form, were developed and implemented within the practices. Participant feedback was obtained through interviews six months after implementation of the resources.

**Results:**

There were 116 participants in the online survey. Management and client care staff (53/116) felt less confident giving owner advice (p<0.01) and recognising critical indicators (p = 0.03) compared to veterinary surgeons and nurses (63/116). Thirteen themes were identified in the survey relating to owner advice; exercise and owner safety were most frequently mentioned, but conflicting guidance was often given. Fourteen client care staff were interviewed. They were confident recognising colic during a telephone conversation with an owner and identified the most common signs of critical cases as sweating and recumbency. The new resources received positive feedback; the decision flow chart and information on critical indicators were identified as most useful. After resource implementation, there was an increase in confidence in recognising critical cases and giving owners advice.

**Main limitations:**

Limited sample population.

**Conclusions:**

This study described existing approaches to telephone triage, identified variations in advice given, and worked with client care teams to develop new resources to aid decision-making.

## Introduction

The process of triage in human medicine was developed to identify and prioritise patients who require immediate medical attention [1]. Telephone triage has become an integral part of modern patient care systems [2]. In human medicine, it is typically performed by trained nursing staff with medical knowledge and communication skills training (Robertson-Steel, 2006). Disease specific protocols or computer-based algorithms, such as the Clinical Assessment System (CAS), are widely employed by triage nurses within the National Health Service (NHS) to support decision-making.

In veterinary practice, animal owners frequently seek professional advice or emergency assistance by telephone. Telephone calls received within a veterinary practice can be managed by a variety of skilled staff [3], including those with little or no clinical training and variable levels of experience. Evidence on use of telephone triage within the veterinary profession is lacking, with existing studies mainly focusing upon clinical triage protocols [4–7] or an owner’s ability to recognise emergencies within small animal practice [8]. In human medicine, telephone triage is integral to the organisation and delivery of emergency care [9–11]. Several standardised protocols have been developed and trialled within human medical setting [12–16], with the effective triage of telephone calls shown to improve patient flow, overall practice workload and reduce financial costs [9, 11, 17]. However, there are currently no standardised telephone triage protocols published within the veterinary literature. This study used a participatory action research approach to develop and implement telephone triage materials for colic (abdominal pain) in the horse in veterinary practice. A mixed methods approach was used to collate information on current approaches in veterinary practice. The researcher then worked with selected practices to explore their experiences and develop and trial new resources. The study focused on colic, as this is the most common emergency problem [18], and a frequent cause of death in the horse [19]. Colic is multifactorial in nature [20–23]; clinical presentation and severity varies between individuals [24, 25] which can make it challenging to recognise signs and identify appropriate actions to take. Early recognition of signs, accompanied by prompt decision-making, is crucial in order to maximise chances of recovery [18, 26], particularly in critical cases requiring urgent surgical or medical intervention [27].

The aim of this study was to describe existing approaches to colic telephone triage in veterinary practice and develop and implement new resources to support decision-making.

The objectives of the study were:

To describe how UK veterinary practice staff triage telephone calls from owners of horses with colic, through an online survey of veterinary practices and interviews with client care / reception teams.
To develop evidence-based resources to support telephone triage in veterinary practice, based on existing evidence on signs of colic, telephone triage systems used in human medicine, and feedback from participating veterinary practices.
To evaluate the implementation of telephone triage resources in participating veterinary practices through structured group and individual interviews with client care / reception teams.

This study met the aims by describing existing approaches and identified inconsistencies in how telephone calls about colic were taken. This included variations in the advice given to the owner and the information passed to the vet, and identified that most variability occurred within the client care teams. New resources, which provided background information on causes and signs of colic, how to identify critical cases, key information to record, and how to ask ‘difficult’ questions, were developed, implemented and evaluated by collaborating with client care teams in four UK veterinary practices.

## Methods and results

The study was reviewed and approved by the University of Nottingham’s School of Veterinary Medicine and Science Ethics Committee. Data collection and anonymity was conducted in accordance with the 1998 Data Protection Act and the British Educational Research Association’s Revised Ethical Guidelines for Educational Research (2004).

The first phase of the research described how staff within UK veterinary practices currently triage telephone calls from owners of horses with colic through an online survey. The second phase developed new evidence-based resources to assist with decision-making during telephone calls about colic in the horse. The approach to telephone triage, as performed by veterinary client care teams, was then assessed, pre and post resource introduction, using structured interviews and questionnaires with client care team members at four UK equine practices.

### Phase 1—Methods

#### Sample population and recruitment for the online survey

The target population for the online survey was veterinary staff who took telephone calls from horse owners during normal working hours. The sampling frame consisted of all UK veterinary practices providing first opinion and referral services for equids.

The survey was distributed by emails sent in July-August 2017 to 787 UK veterinary practices providing equine services, identified using the RCVS ‘find a vet’ online tool. Follow up emails were sent at two and seven weeks post launch. Bounced emails and practices which no longer treated equids were removed from the emailing list. The study was also advertised within relevant veterinary publications (OnSwitch quarterly magazine), on social media platforms (Twitter and Facebook), and flyers distributed at the British Equine Veterinary Association (BEVA) Congress 2017.

#### Development of the online survey

An online questionnaire was developed (SurveyMonkey Inc., California). Questions aimed to investigate participant demographics, approaches to management of telephone calls for colic cases, confidence and perceived difficulties associated with telephone calls of this nature, and whether they were aware of the REACT colic campaign.

The initial survey design was piloted by two equine veterinary surgeons (S1 File). Feedback resulted in the development and inclusion of an additional section focusing on the recognition and management of critical cases, including the use of four clinical vignettes describing differing severities of colic. These were developed by three qualified veterinary surgeons and previously used to assess horse owner knowledge and approach to colic [28]. The final design (S1 File), was piloted by client care team members (n = 5) at a University affiliated equine veterinary practice and two veterinary practitioners (one equine and one companion animal).

#### Data analysis of the online survey

Survey data was filtered (SurveyMonkey Inc., California) to include completed and partial responses from participants who worked in equine practice only. Data was imported directly into an Excel spreadsheet (Microsoft Office 2013, Microsoft) for data cleaning [29], with results updated and saved on a weekly basis.

The mean, median, mode and range were calculated for continuous variables, such as team size and number of years employed within veterinary practice. Normality of distribution was determined using histograms and Kolmogorov Smirnov testing. Mean values were used if data was normally distributed, non-parametric data was reported as the median (range). Categorical and ordinal data were examined for patterns of interest and frequency of occurrence using graphs. Answers which requested a free-text answer were reviewed individually. Answers were then categorised by theme and ranked based on frequency of occurrence.

Categorical and ordinal responses were converted into the appropriate format by the assignment of a number in order to be statistically analysed using SPSS (version 25, IBM Corp. 2017). Due to a small overall sample size, ‘referral hospital only’ and ‘referral hospital with first opinion and ambulatory facilities’ categories were combined, as were the groups’ ‘veterinary surgeon’ and ‘veterinary nurse’ to form a ‘Clinical’ staff group. The category ‘mixed practice’, and associated participant data, was excluded from analysis investigating statistical associations with practice type, due to insufficient sample size (n = 1).

Due to non-parametric distribution of continuous data, a Kruskal-Wallis test with a pairwise comparison using Dunn’s method, was implemented to identify differences between the number of estimated colic calls taken and participant demographics, such as practice type and veterinary team employment. Fisher’s exact testing (FET) was utilised to explore statistical differences between veterinary team employment and colic recognition, case prioritisation, confidence and perceived difficulties. Evidence of association was accepted at p<0.05.

### Phase 1—Results

#### Participants and demographics for the online survey

**Fig 1:**
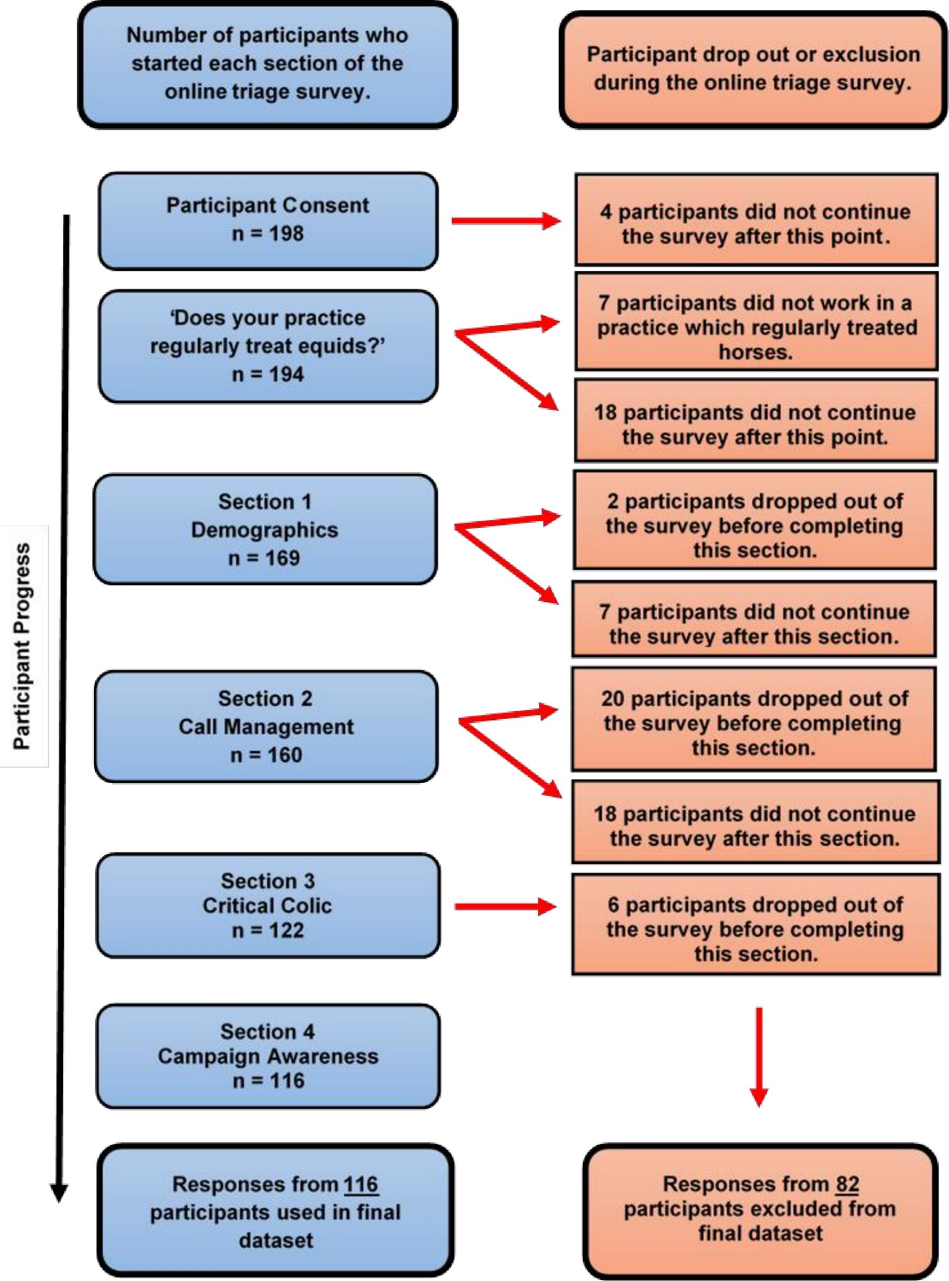
Flow diagram illustrating participant completion of an online survey exploring the telephone triage of colic within UK veterinary practice.

A total of 198 participants consented to participate in the online survey; 82 participants who did not complete more than 50% of survey questions and were therefore excluded from the study (Fig 1). A total of 116 participants were included within the final dataset. Several questions, particularly those requiring free-text answers, were omitted by some participants. The total number of responses (n =) for each question is reported.

A variety of veterinary employees participated within this study: 50% (58/116) were veterinary surgeons, 25.9% (32/116) were client care team, 18.1% (21/116) were management staff and 4% (5/116) were veterinary nurses. Respondents were primarily employed within first opinion equine practice with 51.0% (59/116) operating on an ambulatory basis only and 33.0% (39/116) having both ambulatory and hospital facilities. A small proportion of participants worked within a referral hospital environment (17/116) with only one participant working within mixed (equine and small animal) practice. The ‘REACT’ campaign was recognised by 42.2% (49/116) of respondents. Client care teams had the lowest level of knowledge regarding the project, with 65.6% (21/32) stating they were not aware of the campaign or associated resources.

Participants were employed in teams consisting of a median of five individuals (IQR 3–6 personnel), and had a median duration of 4.5 years (IQR 1.5–12.8 years) employment within veterinary practice. There was no statistical difference in the duration of experience between practice roles.

A median of four telephone calls (IQR 2–10 telephone calls) concerning equine colic were reportedly taken per month by participants. Those employed within a client care role took a significantly higher number of telephone calls associated with colic (median 10, IQR 3–15 calls)) when compared to those employed in a clinical (median 3, IQR 1–5 calls, p = <0.001) role. Clinical personnel managed significantly more telephone calls per month when compared to those in a managerial role (median 5, IQR 3–10, p = 0.043).

Equine referral hospitals took a greater proportion of colic related telephone calls (median 10, IQR 7–18calls) when compared to first opinion ambulatory (median 3, IQR 1–6 calls, p = <0.001) and hospital (median 4, IQR 2–10calls, p = 0.006) practices. There was no significant difference between estimated number of telephone calls between the client care and management teams or first opinion ambulatory and hospital practices.

#### Management of telephone calls in veterinary practices from the online survey

The second section of the survey focused on how participants managed telephone calls about colic. Participants would ask for the owners details (n = 116) and the signs observed (n = 114) in the majority of telephone calls (Table 1). Questions associated with owner access to equine transportation and the current insurance status of the horse were asked less frequently (Table1).

**Table 1:** Topic and frequency of questions asked during telephone triage of colic from an online survey of staff within UK veterinary practices.

Question Topic	Total number of responses	Percentage of telephone calls in which this topic would be raised.
Horse owner details	n = 116	99.0% (range 23–100%)
Signs observed	n = 114	95.0% (range 23–100%)
Duration of signs	n = 113	91.0% (range 0–100%)
Horse signalment	n = 114	64.0% (range 0–100%)
Horse’s current or previous medical history	n = 90	49.0% (range 0–00%)
Owner’s views on referral	n = 64	26.0% (range 0–100%)
Owner access to equine transportation	n = 70	24.0% (range 0–100%)
Insurance status of the horse	n = 64	21.0% (range 0–100%)

Thirty participants, who had submitted ‘0%’ for some question topics, provided further explanation within the optional comments box. The majority (16/30) indicated that details such as insurance, referral options, health history and access to transportation, had been left as ‘0%’ as these would be discussed by the attending practitioner during the visit. Additional reasons for not asking question topics included the knowledge level of the owner (n = 2), the current emotional state of the client (n = 5) and the availability of this information already through a computer database (n = 10).

When participants were asked to rank information obtained in order of importance, owner details (56.0%, 65/116) and signs observed (42.0%, 42/100) were ranked in first and second place respectively. Lowest importance was assigned to the insurance status of the horse (eighth place, 42.1%, 43/102) with referral options (33.3%, 33/99) and access to transportation (30.0%, 30/98) being ranked in sixth and seventh place respectively.

Participants were then asked how they recorded information during a telephone call. The most common method was making rough notes on a notepad before entering details onto a computer-based file (42.0%; 48/116). The use of a specific paper (5.1%, 6/116) or computer (6.9%, 8/116) based form were least favoured. The majority (43.0%, 49/114) of respondents would relay call information via telephone to an ambulatory vet closest to the client, followed by verbally informing an in-house vet (18.4%, 21/114) and both calling an ambulatory vet directly or sending call details electronically to their phone, computer or tablet device (13.2%, 15/114). Eleven participants reported additional methods of relaying information (S2 File).

Participants’ confidence relating to telephone call management varied: 75.9% (88/116) of participants described themselves as very confident in both recognising colic signs and knowing what history a vet would require. When asked about identifying potentially critical cases of colic, 36.2% (42/116) described themselves as fairly confident or not very confident (7.8%, 9/116). The majority of participants (59.5% (69/116) felt very confident in providing advice to clients, but 11/116 participants (9.4%) that were not very confident, and nine of these were working within a client care role. Fisher’s exact testing indicated that confidence levels were significantly different in three areas when compared to participant role within veterinary practice: knowing what history to obtain (p = 0.001), providing owner advice (p <0.001) and overall when taking a telephone call about colic (p = 0.007).

When asked what aspects of a telephone call about colic were difficult, the majority participants responded that knowing what key information to ask an owner was the least difficult feature of a telephone call (94.0% (109/116). Asking an owner potentially difficult questions was perceived as most problematic with 76.5% of participants describing this as fairly (63/115) or very (25/115) difficult. There was a significant difference in perceived difficulty providing an owner with advice and participants role within veterinary practice (p = 0.006).

**Fig 2:**
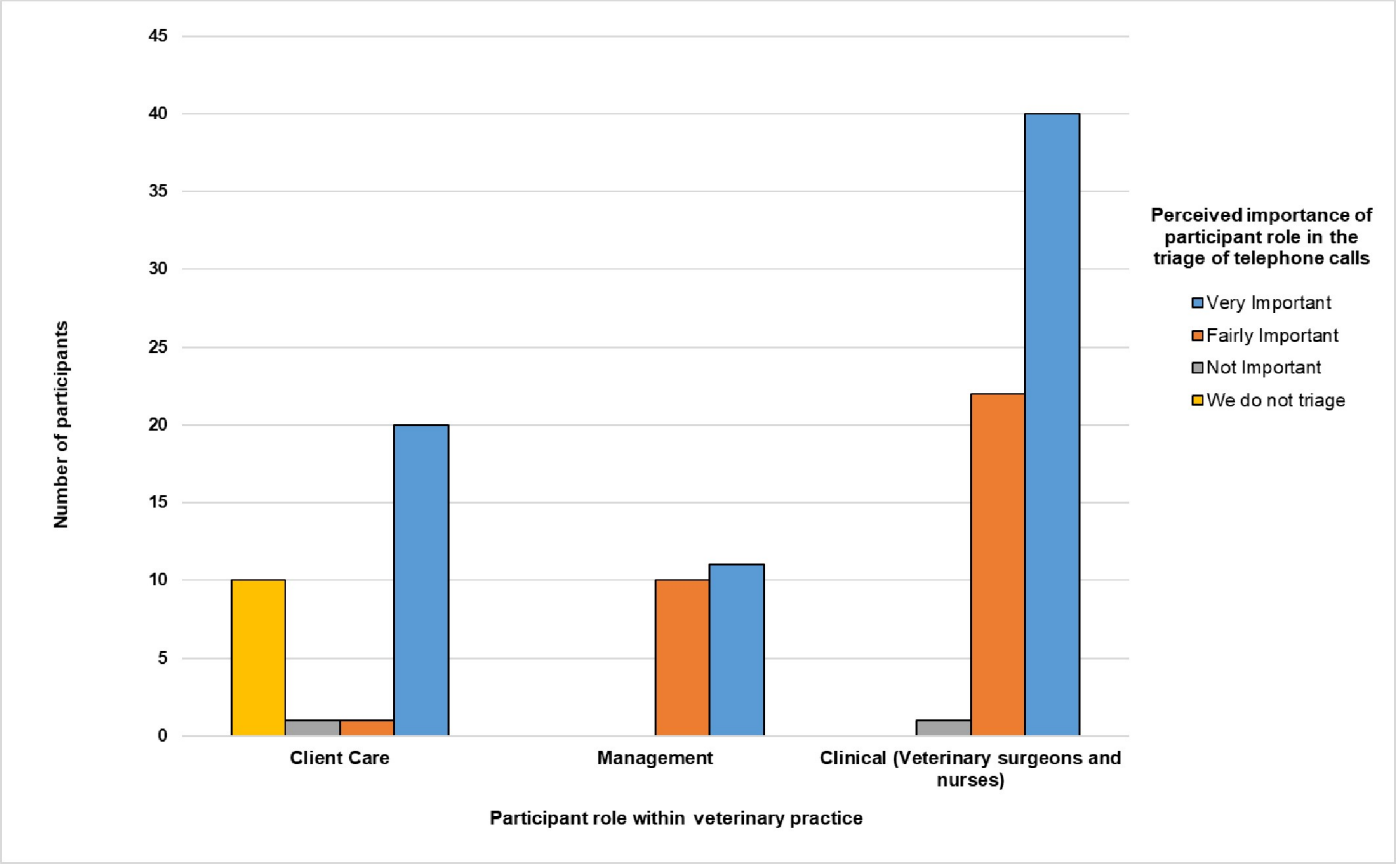
Participant (n = 116) views on the importance of their role in the triage of colic related telephone calls in an online survey exploring current methods of colic telephone triage UK veterinary practice.

The majority (61.2%, 71/116) of participants felt their role in the triage of telephone calls pertaining to potential colic episodes was very important. However, this view varied both across and within individual teams (Fig 2), with ten participants that worked within a client care role indicating that it was not their job to triage calls.

#### Approaches to triage of critical cases of colic from the online survey

The third section of the questionnaire asked about participants’ approaches and knowledge of critical cases of colic, including closed and free text questions.

Analysis of free-text answers (n = 109) to the question *‘Which pieces of information do you feel could indicate a potentially critical case of colic*?’ identified 38 clinical signs participants would associate with critical cases (S8 File). The three most frequently nominated signs were duration of clinical signs (38.5%; 42/109), severity of signs (33.9%; 37/109) and rolling (23.9%; 26/109). Duration and severity of signs were commonly reported together. Four participants, all performing a client care role within practice, suggested that it was not their decision to decide if a case was critical:

*‘As office staff we treat every colic as immediate and the vets make that decision when they speak to the client initially*. *We do not have enough clinical background*.*’* (Survey participant 27).

When presented with Scenarios one and four, (in which colic was not specified), there was variation in participant views on whether abdominal pain was suspected. Most participants (65/115) felt it was fairly likely that Scenario one (case with mild clinical signs) described a horse displaying signs of colic, followed by 19.1% (22/115) stating it was very likely, and 14.8% (17/115) not likely. When presented with Scenario four (case with severe/critical signs), 54.8% (63/115) indicated that it was very likely, 33.9% (39/115) fairly likely and 3.5% (4/115) not likely to be displaying signs of colic. There was no statistical difference between recognition of colic and role within veterinary practice.

Scenario three (severe/critical case in which colic was specified) received the highest priority overall with 97.4% (110/113) of respondents reporting that this case would be prioritised as highly urgent. Scenario one (mild/medical episode in which colic was not specified) was given lowest priority, with 77.6% (90/116) assigning medium urgency to this case. Participants who worked within client care teams appeared to be most unsure, with 21.9% (7/32) indicating that they did not know what level of priority to assign to Scenario one.

Analysis of free-text responses for all four Scenarios to the question, *‘Are there any other questions you would ask the owner about themselves or their horse during this initial phone call*?*’* identified 20 main areas of questioning. Ascertaining the exact location of the horse, whether the horse was showing normal behaviour, and previous/current medical history were the questions most frequently asked (Table 2). Questioning varied between Scenarios, with participants referring to duration of signs and owner preparation, such as organising transportation, more frequently when presented with Scenarios two (mild/medical episode) and three (severe/critical case).

**Table 2:** The top six categories identified during free-text analysis of answers associated with the question *‘are there any other questions you would ask the owner about themselves or their horse during this initial phone call*?*’* in an online survey exploring current methods of colic telephone triage in UK veterinary practices.

Question Topic	Number of participants reporting question topic for each scenario				Total times topic reported
	Scenario 1 Mild / Medical colic (e.g. impaction)	Scenario 2 Mild / Medical colic (e.g. spasmodic)	Scenario 3 Severe / Critical (e.g. strangulating lesion)	Scenario 4 Severe / Critical (e.g. grass sickness	
Where is the horse located?	20.8% (20/96)	29.5% (26/88)	24.1% (19/79)	22.7% (17/75)	82
Is normal behaviour being shown?	19.8% (19/96)	18.2% (16/88)	22.8% (18/79)	20.0% (15/75)	68
What is the horse's previous/current medical history?	21.9% (21/96)	17.0% (15/88)	10.1% (8/79)	26.7% (20/75)	64
Horse details (age, insurance)	10.4% (10/96)	14.8% (13/88)	19.0% (15/79)	13.3% (10/75)	48
How long has the horse been showing signs?	6.3% (6/96)	25.0% (22/88)	Not reported	17.3% (13/75)	41
What signs is the horse showing?	30.2% (29/96)	3.4% (3/88)	1.3% (1/79)	4.0% (3/75)	36

Analysis of free-text responses for all four Scenarios relating to the question, *‘Would you give the owner any advice on what to do whilst they waited for the vet to arrive*?*’* identified 17 advice topics (S9 File). Advice regarding safety, horse exercise and the removal of food was most frequently suggested (Table 3). Exercise was commonly referred to as walking the horse ‘in hand’ however, duration of exercise varied with some participants often implying it should be done until the vet arrived, whilst others simply stated, ‘walk the horse’. The most frequently mentioned safety aspects were the removal of potential hazards, such as loose buckets or haynets, and advice that the owner should stay at a safe distance from the horse.

**Table 3:** Top six categories identified during free-text analysis of answers associated with the question *‘Would you give the owner any advice on what to do whilst they waited for the vet to arrive*?*’* in an online survey exploring current methods of colic telephone triage in UK veterinary practice.

Advice Topic	Number of participants reporting advice topic for each scenario				Total times advice topic reported
	Scenario 1 Mild / Medical colic (e.g. impaction)	Scenario 2 Mild / Medical colic (e.g. spasmodic)	Scenario 3 Severe / Critical (e.g. strangulating lesion)	Scenario 4 Severe / Critical (e.g. grass sickness	
Horse and owner safety	11.7% (12/103)	53.3% (57/107)	47.3% (44/93)	19.0% (16/84)	129
Horse exercise	31.1% (32/103)	36.4% (39/107)	16.1% (15/93)	15.5% (13/84)	99
Removal of feed	29.1% (30/103)	15.9% (17/107)	7.5% (7/93)	10.7% (9/84)	63
Monitor the horse	24.3% (25/103)	7.5% (8/107)	9.7% (9/93)	8.3% (7/84)	49
Not allowed to provide advice	9.7% (10/103)	9.3% (10/107)	10.8% (10/93)	11.9% (10/84)	40
Would not give advice for this scenario	11.7% (12/103)	2.8% (3/107)	8.6% (8/93)	13.1% (11/84)	34

Advice varied between Scenarios with participants referring to safety aspects more frequently when presented with Scenarios two (mild/medical episode) and three (severe/critical case), whereas Scenario one (mild/medical episode) was most commonly associated with the removal of food. Contradictory advice was noted on several occasions, especially when referring to whether the horse should roll:

*‘Walk the horse lightly around the yard*, *try to avoid the horse from rolling*.*’* (Scenario 2, survey participant 113)

*‘Try to prevent horse rolling by walking around arena in hand*.’ (Scenario 2, survey participant 108)

*‘Leave horse in stable*, *take-out food*, *water bucket etc*., *and allow to roll if it wants—safety concern for owner if in stable*.*’* (Scenario 2, survey participant 60)

*‘Nothing specific to do in this case*, *leave be in the stable and allow to roll*.*’* (Scenario 2, survey participant 66)

Advice would frequently not be given to owners, with ten participants, all undertaking a client care role within practice, stating that this was not their responsibility:

*‘Not allowed to advise as non-clinically trained personnel*.*’* (Survey participant 12)

*‘We would get a vet to give advice as we are unable to give advice’* (Survey participant 14).

### Phase 2—Methods development and piloting of new resources on telephone triage

Materials were developed to provide current information about equine colic and support decision-making by those routinely taking telephone calls from horse owners. The resources developed were an evidence-based information document on colic, a recording form to document information during telephone calls about colic, and a decision-making flow chart. The written document consisting of the following advice sections: ‘Does the horse have colic?’, ‘Is the case likely to be critical?’ and ‘What should the owner be doing?’, based on information disseminated in the ‘REACT’ colic campaign (www.bhs.org.uk/colic). Information presented within the materials was based upon evidence reviews, multi-stakeholder surveys/workshops, expert opinion pieces and evidence from human medical literature (Table 1). The preliminary design was piloted amongst a small group of equine veterinary staff (receptionists, nurses, administration and management) (n = 8) at Cx Congress (Nottingham, UK), and.professionals (n = 8) working within the education department at the School of Veterinary Medicine and Science. A summary of the final format, following feedback from the pilots is described in Table 4.

**Table 4:** Sections, and associated content, included within the initial design of the telephone triage resource for veterinary practices on colic in the horse. Reason for inclusion and source of evidence is listed.

Section	Overview of Content	Reason for Inclusion	Source of Evidence
***Resource Introduction***	Introduction to resource content and purpose. Specific reference to RCVS Practice standards scheme, the Vet React website and ‘REACT’ campaign.	Inform practices how the materials can be used to satisfy module 3 of the RCVS Practice Standards Scheme (Protocol for recognising and dealing with requests for emergency treatment) and direct them to further resources.	RCVS Practice Standards Scheme: www.rcvs.org.uk/setting-standards/practice-standards-scheme
***‘Does the horse have colic*?*’***	Overview of colic and commonly reported clinical signs.	Increase awareness of subtle signs owners may overlook. Advice on how to gather key information that can assist the attending practitioner and encourage owner preparation.	Multi-stakeholder workshops, online horse owner Delphi consensus and survey of horse owner knowledge of equine colic [28, 30, 31]
***‘Is the case likely to be urgent/critical*?*’***	Overview of potential critical indicators and examples of targeted questions that can establish level of urgency.	Increase awareness of potentially critical cases that require immediate medical or surgical intervention.	Retrospective and prospective studies of horses presenting with abdominal pain and multi-stakeholder workshops [18, 30, 32]
***‘What should the owner be doing whilst waiting for the vet to arrive*?*’***	Standardised advice for owners waiting for the vet to attend.	Practical advice which can be given to ensure the safety of both horse and owner whilst the vet is on route.	Recommendations based upon expert opinion (Professor Deborah Archer BVMS PhD CertES(Soft Tissue) DipECVS MRCVS)
***‘Approaching the difficult questions’***	Overview of potentially sensitive topics that may be discussed during a colic telephone call.	Guidance on phrasing can be used to create opportunities for further discussion. Written examples of targeted questions also included	Aspects of human medical literature focusing on communication skills for general practitioners (GP’s) (Silverman et al., 2016) and key findings associated with colic recognition and decision-making [30, 31]
***Recording Form***	Paper-based form to record information gathered during a telephone call.	Bespoke form that can be used by practice teams to collect key information in a logical manner during a telephone call about colic.	Sections based upon information discussed within the triage resource and elements suggested within veterinary literature [33]
***Decision-making Flow Chart***	Flowchart displaying information which should be collected during a telephone call about colic.	A quick reference resource that practice teams can refer to during a telephone call to ensure key information is gathered,	Elements are based upon pack information an structure suggested by the Schmitt and Thompson office-hours telephone triage protocol (www.cleartriage.com)

#### Sample population for the client care team interviews pre and post resource implementation

The target population for this second phase were members of client care teams (those working front of house / on reception only) currently working within veterinary practices, which provided first opinion services for equids on a daily basis. A convenience sample of four client care teams working within East Midlands veterinary practices were recruited. Practice profiles are detailed in S3 File.

#### Assessment of telephone triage of colic cases by client care teams pre and post resource implementation

Structured group interviews, using a verbally administered questionnaire [34], were conducted with each participating client care team. Participants were interviewed prior to introduction of the telephone triage resources and again after a 6-month trial period. A follow-up visit, 10 to 15 minutes in length, was made to each practice three months after resource introduction to further encourage study participation and use of the materials.

The first interviews were conducted by the principle researcher in groups within a formal-field setting (practice reception) [35]. This interview was performed in a three-stage format: 1) verbal questionnaire administration, 2) introduction of telephone triage resource, 3) completion of feedback forms. Participants were left with multiple copies of the telephone triage resources for further use. The second interviews, 6-months post resource introduction, were conducted in groups or on an individual basis via the telephone, depending on the practice circumstance and preferences.

Interview responses were recorded verbatim by hand using a standardised form. Additional field notes were also maintained. Question responses were the main consensus of the entire group after a short discussion, except for those aimed specifically at individual confidence, or responses gathered on an individual basis via the telephone.

Questionnaires for both interviews were quantitative in structure using pre-determined questions [36, 37]. Where open questions were employed, only single word answers or short descriptions were required. Questions within the initial verbal questionnaire were developed following group discussion with researchers and qualified veterinary surgeons at the School of Veterinary Medicine and Science (n = 7). Areas covered were current methods of call management, knowledge of potential critical indicators, call difficulties and overall confidence when handling queries associated with colic.

The questionnaire schedule was piloted by three veterinary surgeons and included a scenario describing a potential telephone call about colic, to ensure participants understood which type of call the questions referred to, followed by nine questions of varying types (Table 5). Question four, which was directly associated with personal confidence and demographics, was presented on a separate document along with pre-defined answers for participants to complete individually (S4 File). Initial feedback directly associated with the telephone triage resource was collected using a paper-based form listing six pre-determined questions (S5 File) during the first interview session.

**Table 5:** Questions and scenario implemented within a verbal questionnaire for client care staff within four UK veterinary practices prior to introduction of resources to support decision-making in telephone triage of colic.

*Scenario*: *An owner telephones the practice as they think their horse may have colic*. *They are quite emotional and worried about their horse and would like a vet to visit them as soon as possible*.		
Question	Question Type	Reason For Inclusion
1. How would your team initially manage a call like this?	Open	Establish current methods of telephone triage.
2. Which three pieces of initial information do you feel are most important?	Open	
3. During the telephone conversation, how is the information the owner gives recorded?	Open	
4. After the initial conversation, how is a potential colic case reported to the rest of the practice team?	Open	
5. As a group, what signs do you think relate to a potentially critical case of colic?	Open	Establish current views of colic and identifying gaps in knowledge.
6. When talking to an owner over the phone, how confident, as a team, do you feel about– Recognising critical cases Knowing what information a vet will need About giving an owner advice Overall when taking a call about colic Recognising signs of colic (Pre-defined answers: Very confident, Fairly confident, Not very confident)	Rating of confidence	
7. Reflecting on past experiences, what is the most difficult aspect of taking a call about a possible colic case?	Open	Establish areas for further support.
8. Triage is defined in the Oxford Dictionary as: ‘The assignment of degrees of urgency to wounds or illnesses to decide the order of treatment of a large number of patients or casualties’ Regarding a potential telephone call about colic, how important do you view your role in the triage of these calls? (Pre-defined answers: Very important, Fairly important, Not important, We don’t triage that’s the job of the vet)	Closed with pre-defined answers	Establish views and concerns in relation to telephone triage.
9. Are you aware of the ‘REACT’ colic campaign?	Closed with pre-defined answers Yes/No	Establish awareness of previous research and campaign

The second questionnaire, conducted six months post resource introduction, aimed to reassess participants’ approaches to call management, and establish whether the triage resources had been used within the practice.

A total of ten questions, with associated ‘prompts’, were included in the final questionnaire design (S6 File). The final questionnaire was piloted by two veterinary surgeons. Questions one to four were taken directly from the first questionnaire to enable direct answer comparisons. Questions five to ten investigated use of the telephone triage resource, awareness of the ‘REACT’ campaign and the potential impact of these factors on knowledge and behaviour. An individual form was again distributed to participants for question two, which referred directly to personal confidence.

The primary author of this research study (Katie Lightfoot) organised and performed all aspects of data collection and subsequent analysis. An in-depth reflexivity statement for this researcher is available in S7 File.

A researcher-participant relationship was generally not established prior to data collection, with study participants only contacted in order to arrange a mutually convenient interview date and time. Participants were provided with an information sheet describing the purpose of this research study when invited to participate.

#### Data analysis for the client care team interviews pre and post resource implementation

Practices were randomly assigned a number between one and four to anonymise responses. The individual forms describing personal confidence were assigned a single letter in order to identify and compare answers from both interview sessions. Responses were manually input into Excel (Microsoft Office 2013, Microsoft).

Descriptive analyses were performed on quantitative data (mean, mode and range) relating to group size and years’ experience within veterinary practice. Answers to closed questions (both within the questionnaire and resource feedback form) were ranked based on frequency of occurrence. Qualitative responses were manually sorted into categories (for example, behavioural signs of colic, method of recording client details)by the principle researcher (KL) and ranked based on frequency of occurrence. Categories were reviewed by a secondary researcher (HC) to identify potential issues and refine codes. In line with Morse (1997), no further coding was performed by the secondary coder. Additional statements and short phrases captured within the interviewer’s field notes were used to support participant responses where appropriate.

### Phase 2—Results

#### Interview setting and participant demographics

The interviews before resource implementation were performed at participating practices between June and August 2017. Sessions had a mean duration of 52 minutes (range, 45–60 minutes) and were conducted in the reception space at Practices One, Three and Four, and within a formal meeting room at Practice Two.

The interviews after resource implementation were conducted between January and February 2018. These sessions had an average duration of 16 minutes (range, 11–26 minutes) and were performed in the reception space at Practice Four, within a formal meeting room at Practice Two and via the telephone on an individual basis with participants at Practices One and Three.

In total 14 client care team members, with interview group sizes ranging from two to six individuals, participated in this study. Client care teams at Practices One and Three were each represented by two members of staff, whereas groups of four and six personnel agreed to take part at Practices Two and Four respectively.

Overall, participants had a meanof 11.1 years (range, 0.16–40 years) experience working within veterinary practice. All participants had personal equine experience, including horse ownership and previous employment within the equestrian industry.

All client care teams had a multi-faceted role within practice, which included telephone call management, organising owner-requested prescriptions (i.e. worming products), taking payments and the processing of insurance claims. Participants at Practices Three and Four managed calls relating to equids only, whereas the remaining practices provided initial contact for owners of both equids and either companion animals (Practice One) or livestock (Practice Two).

#### Telephone call management by client care team interview participants

The following interview findings address four main aspects explored within this study. The first provided an overview of participant demographics, including equine and veterinary practice experience. The second, ‘telephone call management within practice’, identified how owner-reported calls of equine colic were managed prior to resource introduction, and compared this with participant responses after the 6-month trial period. The penultimate section, ‘confidence and potential call difficulties’, compared participant reported confidence levels relating to various aspects of telephone call management, before and after resource introduction, and what signs teams would consider to be indicators of cases in need of urgent veterinary attention. In the final section, participant views on the potential impact of the telephone triage resources on their approach to call management, and awareness of the REACT to beat colic campaign, are described. Responses are presented as the main consensus of each practice team (i.e. Practice One), with words or phrases directly quoted (displayed in quotation marks), by individual participants (i.e. Practice One, participant A), used to illustrate key points.

During the first group interviews, three practice teams stated that an initial call about equine colic would be managed by taking a detailed history from the owner. Those employed at Practice Two stated that a basic history, encompassing just owner contact details and nature of the complaint, would be obtained before informing a vet. All practice teams made direct reference to recording both the signs currently being shown by the horse and the duration of the episode. Establishing the best contact number for the owner was stressed by all teams with past issues relating to ‘drops in telephone signal’ and ‘calling from a friend’s phone’ alluded to. Additionally, all teams made recurring reference to ensuring the right location of the horse was obtained, for example, participant A at Practice Four suggested that owners of horses with colic often get in a ‘flap’ and give their home address or simply state ‘a field in (town name)’, instead of providing their correct location. Addressing the emotional state of the owner was referred to several times by teams at Practices Two and Four with comments such as ‘I’d try and keep the owner calm’ stated. When revisited during the second interviews little difference was noted in regard to initial call management, with all four teams again stating that the location of the horse, signs observed, and owner contact details were most important to obtain.

Factors that could potentially affect questioning were noted during the first interviews before resource implementation. Participant A at Practice One suggested that using a computer database most likely influenced their questioning process, as they checked client details ‘starting with what appeared on the computer screen first’. All practice teams referred to how the ‘flow’ of the conversation, and therefore subsequent questioning, would often depend on who the caller was. Those at Practice Four suggested that if they were familiar with a client, they would ask limited questions as to not ‘annoy’ or ‘frustrate’ the caller. Participants at Practice One implied that when talking to an owner you get a ‘feel’ for which questions are ‘appropriate at that time’, with participant B remarking that discussing payment can sometimes ‘upset’ clients or cause them to become ‘abusive’.

A difference in approach to recording information was observed during the first interview sessions with Practices Two and Three opting to enter information directly onto a computer, whereas the remaining two teams preferred to make rough notes on paper first before transferring the information onto the computer. However, when re-evaluated during the second interviews after resource implementation, all teams indicated that an exclusively computer-based approach was preferred. One participant (Practice One) did allude to the occasional use of hand-written notes. Responses during the first interviews revealed that all four teams relay call information via the telephone to an ambulatory vet closest to the owner. Teams at Practices Two and Three did indicate that if it wasn’t possible to contact an ambulatory vet, the in-house practitioner would be informed to ‘prevent delays’. In addition to relaying information over the telephone, it was common practice for key information, such as owner name, location and contact number, to be sent by ‘text message’ to the attending vet by team members at Practice Two.

#### Confidence and potential call difficulties identified by client care team interview participants

When asked about their role in the triage of emergency calls associated with colic, all those participating felt they were very important as they were an owner’s ‘first point of contact’. However, all teams acknowledged that the diagnosis of a case was the ‘sole responsibility’ of a veterinary practitioner.

When explored during the first interviews, dealing with owners of heightened emotion was perceived as the most difficult aspect of telephone communication by all practice teams. Locating an available vet and providing an owner with an ‘accurate timeline’ of when the vet would arrive were further difficulties experienced by Practices One and Two, as this often caused owners to become ‘aggressive’ or ‘impatient’.

When asked about individual confidence during the first interviews, 71% (10/14) of participants reported feeling very confident that they could recognise the signs of colic during a telephone call with an owner. Assurance in this aspect remained unchanged at the second visit with the majority (11/14) still feeling very confident. A slightly lower confidence level was associated with the recognition of ‘critical’ indicators during the first interviews, with the majority (7/14) of participants being fairly confident. At the second interviews after resource implementation, three participants (two at Practice Four and one at Practice Two) reported feeling very confident (compared to ‘fairly’ previously) and one respondent (Practice Four) with lowest confidence previously, now felt fairly confident.

Owner advice received the lowest confidence rating overall during the first interview with only 28% (4/14) of participants feeling very confident in this aspect. Increased confidence in this area was seen at the second interview after resource implementation, with the majority of participants reportedly feeling either very (6/14) or fairly (7/14) confident. One team member (Practice Four) still did not feel confident in this aspect.

**Fig 3:**
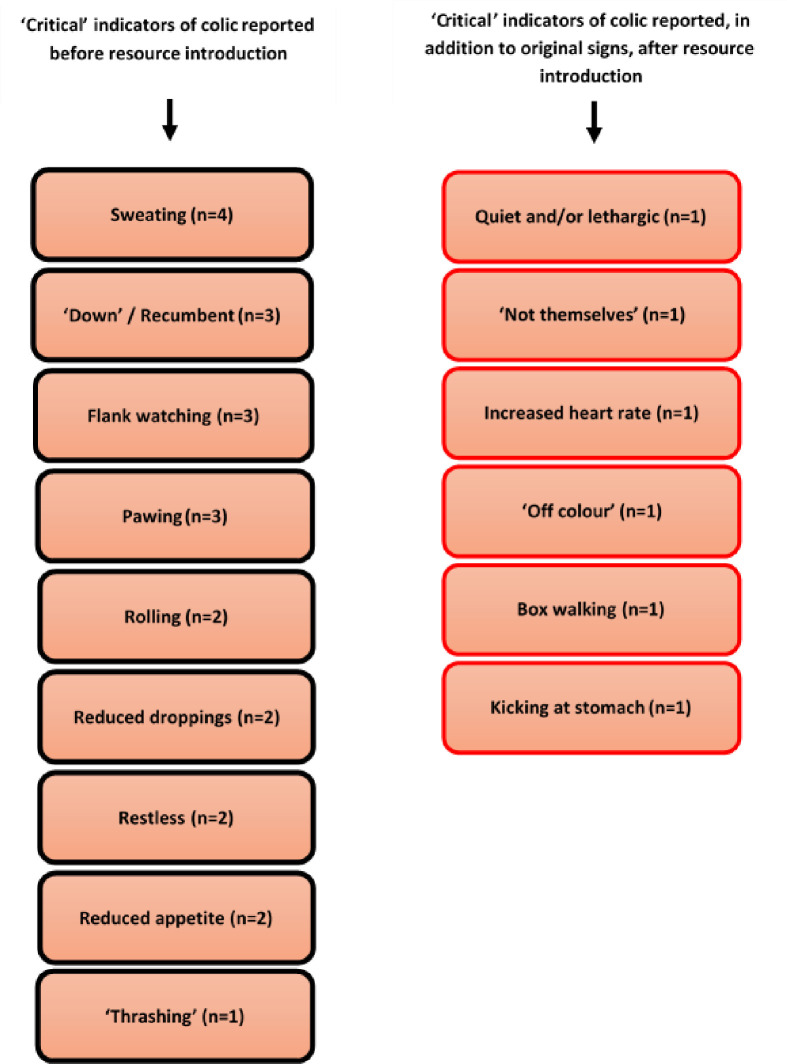
Potential indicators of critical colic reported by participants (n = 14) before (left) and after (right) the introduction of supporting resources in a pilot study exploring the telephone triage of colic calls with UK veterinary practices.

A total of nine potential signs of critical colic were suggested by teams during the first interview, with ‘sweating’ being a key sign mentioned by all practices. Although all nine signs were again reported during the second interview, Practices Two, Three and Four also referred to additional signs, such as lethargy or the horse looking ‘off-colour’ (Fig 3).

#### Resource feedback and use by client care team interview participants

When introduced during the first interviews, all participants indicated that the information pack provided was interesting and relevant, with the sections on ‘Critical signs’ and ‘Approaching difficult questions’ particularly favoured. Practices One and Three suggested that the materials would be especially relevant for staff whose role on reception is ‘split’ between companion animal and equine work.

When revisited during the second interviews, six participants felt the information pack had been very useful with the most popular sections being ‘approaching difficult questions’ and ‘advice to owners’. Additionally, the team at one practice particularly highlighted the section focusing upon critical signs of colic, with participant D commenting that, ‘it makes you recognise signs you would have never associated with colic before like the horse just being quiet’. In spite of this, the majority (8/14) of participants felt the telephone triage pack had not impacted upon the way they approached or managed a colic related telephone call. Interestingly, staff at one practice stated that, although the resource was very useful, it was practice policy for all colic to be treated as potential emergencies and non-clinical personnel ‘were not to give owner advice’.

All participants initially felt that the triage flow chart would be useful as it was clear and logical in design. Most felt that it would be particularly relevant for ‘non-horsey’ or ‘inexperienced’ staff, but more experienced team members would also refer to the chart to ‘ensure all information is taken’. This positive view was echoed during the second visit with six participants actively using the resource during telephone calls. Participant B at Practice Two found the inclusion of key owner advice particularly helpful, stating they now incorporate this into their conversation with an owner and were aware of colleagues doing the same. Those not using the resource cited various factors, such as the ‘way the practice is run’ (Practices One and Four) and ‘reliance on personal experience’ (Practice Three), as reasons for not incorporating the flow chart into their approach. It was again suggested that this resource would ‘mostly benefit those working within mixed practice’.

On the initial interviews, four participants indicated that the colic recording form wouldn’t be used, however, two of these participants (at Practices One and Four) suggested it may be useful as a personal ‘prompt’ to ensure the vet received as much ‘relevant information’ as possible. Some participants felt it would be too long to go through when ‘an owner may be upset,’ (Practice Two) and that some of the information, such as current medication, would be ‘asked for by the vet during the visit’ (Practice Four). When reassessed, none of the 14 participants had used the recording form during the six month trial period. Two participants (Practices One and Two) stated they had used the form as a ‘reference tool’ but had not actively filled it in. Reasons for not implementing this resource included ‘time pressures’ when working on reception (all teams), ‘individual practice suitability’ (Practices One and Three) and a person’s ‘stubbornness to change’ (Practice One).

None of the 14 participants were aware of the ‘REACT to beat colic’ campaign or associated resources during the first interviews. When awareness was re-evaluated during the second interviews, the majority (11/14) of participants felt the resource had not impacted upon their approach. However, several participants (at Practices Two and Four) stated that they had the ‘REACT’ quick reference guide available on their desk to ‘refer to during a call’. Additionally, all practices said they now provide clients with access to ‘REACT’ resources through practice welcome packs or colic education evenings. The majority (12/14) of participants had not noticed any change in the way owners reported colic since being aware of the campaign. Two participants (Practices One and Four) said that they may have noticed a slight change in owner reporting, although they suggested that this may be due to their ‘own increased awareness’ of the condition.

## Discussion

### Summary of findings

Telephone communication is the primary form of contact between owners and their veterinary practice in the event of an emergency. This study was the first to investigate the impact of resources on decision-making during an initial telephone call from an owner of a horse with colic. In the author’s opinion, the results show that the telephone triage of colic cases, and the identification of potentially critical cases, is currently unstandardised within UK equine practice. Information that could potentially impact delays in treatment, such as the insurance status of the horse and access to equine transportation, were rarely queried during an initial telephone call with an owner. Though Client care, rather than clinically-trained employees managed the majority of telephone calls within practice, some reported that they felt unconfident providing advice to owners and identifying potentially critical cases. Resources to support decision-making in the telephone triage of colic were developed based on existing evidence and health campaigns [18, 30–33], and systems used in human telephone triage [16]. The resources were viewed positively by client care team, but factors such as computerised booking systems, owner familiarity and practice protocols were potential barriers to implementation. Some staff were more confident in recognising critical cases and giving advice to owners after introduction of new resources.

### Study limitations

This study utilised a mixed-methods approach to data collection. Recognised as the third research paradigm [38, 39], mixed-methods research draws from the strengths of both quantitative and qualitative methodologies. As a result, phenomena can be explored from a variety of perspectives [38].

The results of this study cannot be considered representative of all UK veterinary practices, as they are likely subject to sampling and selection bias. The sample sizes obtained in both phases of this study were small therefore, interpretation of findings, trends, or differences in practice approach to triage cannot be reliably determined at this stage. Additionally, it must be highlighted that this study focused on the theoretical triage of a telephone call pertaining to colic. Therefore, inferences relating to the impact of individual approaches to telephone triage on case outcome was not explored.

The use of convenience sampling to recruit interview participants may have led to the collection of data specific to individual practice environments, though given opinions were consistent across all practice teams. Due to time pressures associated with data collection and barriers encountered during practice recruitment, such as a reluctance to participate outside of normal working hours, structured group interviews were chosen as the most appropriate method of data collection for this study. However, it is acknowledged that may have resulted in some participants being more inclined to give answers they considered socially acceptable amongst their peers. Additionally, sessions were not audio recorded due to the fact nearly all interviews were performed within the reception space, which may have inadvertently led to the recording of sensitive client information.

Survey distribution was facilitated using targeted emails, social media dissemination and journal advertisement, yet the overall response rate was low. Although inclusion criteria was clearly explained within both the email invitation and survey introduction, the majority of respondents were qualified veterinary surgeons with high levels of item non-response noted. Though disappointing, this finding reflects the growing issue of survey nonresponse in social science research [40], particularly when investigating professional roles, such as those of General Practitioners (GPs) and nurses [41–45]. Additionally, those working within a client care role are difficult to access directly, which may account for low study engagement within this group.

### Key findings

The telephone is a predominant form of communication within the veterinary industry [46], with those frequently managing telephone calls in practice being the first point of contact for owners requiring emergency advice [47]. It was not surprising that the majority of colic related telephone calls within this study were initially taken by those working in a client care role. Historically referred to as ‘receptionists’, these team members are often considered gatekeepers to veterinary care [48] and are an integral part of the customer care experience [49]. Yet, despite being a client’s first line of assistance, conflicting views on their role in the telephone triage of colic were noted. For instance, when responding to the online survey, 62% of those employed in a client care role felt they were very important in the triage of calls, yet a further 30% suggested that this was not their responsibility. A similar concept has been noted within the socio-spatial environment of human patient care. In a role not so dissimilar to that of client care teams, General Practice Receptionists (GPRs) have been found to disagree on their position in the triage of patients seeking access to primary care. When performing qualitative interviews with 14 GPRs, Neuwelt *et al*., (2015) observed that whilst some participants felt that it was definitely their responsibility to ascertain patient eligibility for medical appointments, others were adamant that this role was the sole responsibility of the General Practitioner (GP) or nurse. Interestingly, despite a conflict of opinion, all GPRs participating indicated that they would perform varying degrees of informal patient triage when health resources, such as medical consultations, were in high demand. Congruent with these findings, Alazri *et al*., (2007) noted that receptionists often rely on their limited medical knowledge to ascertain call severity, with decision-making significantly influenced by practice culture, systems and policies. In the current study, client care members participating in group interview sessions indicated that their questioning was often influenced by client familiarity, assumed knowledge or emotional state. Based on this information, it is reasonable to suggest that client care teams may inadvertently perform some degree of telephone triage, despite role uncertainty. These results highlight the need for industry stakeholders to recognise the complex, and often unsupported, role that client care teams perform in veterinary practice. As the first point of emergency contact for animal owners, conditions within the socio-spatial environment of reception can often be challenging for client care personnel. Therefore, more should be done to ensure these team members are sufficiently supported during times of uncertainty, through the implementation of formal training and standardised protocols. The decision-making ‘flowchart’ described during this research project can provide client care teams with an essential questioning framework that can be used to support decision-making when confronted with a potential emergency. Additionally, providing these key staff members with standardised, non-clinical client advice, will ensure that all clients will receive similar congruent advice aound the safety of both horse and owner until the arrival of a veterinary professional.

The multi-factorial nature of colic can present many challenges [21], with differentiation between critical and non-critical cases presenting further quandaries for attending practitioners. For critical cases, identified as horses requiring hospitalisation for intensive medical or surgical intervention [32], rapid recognition is vital to securing a successful outcome. The findings of this study suggest that veterinary staff managing initial telephone calls assess cases based solely on verbal information provided by the owner and advise the attending veterinary surgeon accordingly. Veterinary staff are therefore relying on an owner’s interpretation of signs and knowledge of when the horse was last seen to be behaving ‘normal’ in order to assess case severity. However, confidence in the recognition of potentially critical cases was low within this study population, with very few responses in the online survey identifying potential indicators of critical colic, with many frequently referring to the ‘severity’ or ‘duration’ of symptoms instead. However, it is important to note that 50% of participants were veterinary surgeons, who reported higher levels of confidence in both interpretation of reported colic signs and owner communication. This may have resulted in client care teams appearing disproportionately less confident when compared to those with specific clinical training. Nevertheless, previous studies have identified that horse owners may under-report clinical signs [50, 51] and have difficulty recognising potential ‘red-flag’ indicators of critical cases [28]. Therefore, it is imperative that veterinary staff answering telephone calls are able to establish complaint severity and prioritise cases accordingly [47].

In order to achieve General Practice accreditation, establishments providing equine services must ensure that all team members responsible for answering the telephone are trained to recognise potential emergencies, such as colic [52]. Additionally, employees should be effective in the prioritisation of cases requiring immediate veterinary treatment. Therefore, it was interesting to note that a small proportion of those within client care roles were unsure what priority some colic scenarios should be assigned. Although this could be attributed to the fact that several of these personnel suggested that it was not their responsibility to assign case priority, this finding warrants further investigation.

Advice for owners of horses with colic was varied, although it was reassuring that the majority of participants frequently referred to horse and owner safety. Conflicting advice, particularly associated with allowing the horse to roll, was highlighted. Exercise as a means of preventing a horse with colic from rolling is frequently performed by owners [24, 28] due to the misconception that rolling increases the risk of the intestinal tract ‘twisting’. Although controlled exercise may be beneficial in some circumstances [53–55], it should only be performed under the supervision of a qualified veterinary surgeon. The fact that this study highlights that owners may receive potentially conflicting advice from veterinary staff regarding this topic is concerning. The role of client care personnel is not regulated within the veterinary industry, nor is there a requirement for these individuals to obtain a formal qualification in order to perform the vast array of tasks asked of them [56]. However, it is recommended that employees who frequently manage telephone calls within veterinary practice should be adequately trained in the triage of potential emergencies, including the provision of basic client advice [47]. Previous research has indicated that both pet owners and veterinary surgeons perceive the client care team as a source of both preventative [57] and pet health advice [56, 58], although these studies focused on small animal employees rather than those in equine practice. Practices should have processes in place to provide training and support for staff giving advice and involved in decision-making. The resources developed during this study should assist with this training and support.

Resources designed to support decision-making were initially viewed positively by client care teams, yet many felt that the materials had not impacted upon their approach to the telephone triage of colic. This was in contradiction to some participant’s suggestion that they found elements of the information pack, such as guidelines on how to approach difficult questions, very useful. The use of group interviews may have introduced peer pressure and reluctance to discuss perceived knowledge gaps or concerns. Conversely, time-pressure during a telephone call about colic was referred to by all interview participants, which could indicate that, despite some perceiving the resources to be useful for personal development, it is not practical to refer to them during an actual call. Other barriers were also identified: some interviewees indicated that current ‘practice policy’ did not allow them to utilise resource information, such as providing basic advice, during telephone calls with clients. Although policy-makers within practice may discourage non-clinical staff from providing ‘clinical’ advice to clients on the assumption that they have not been trained to do so, this lack of guidance may result in staff members inadvertently relying on moral judgement or prior experience to fill gaps in knowledge during pressured and uncertain conditions. In comparison, non-clinical members of staff are becoming frequent triageurs of urgent calls within human medical establishments. For example, in 2013, the National Health Service (NHS) launched the NHS 111 initiative, which specifically employs non-clinical call handlers to triage urgent patient calls [59]. However, unlike veterinary client care teams, NHS 111 call handlers undergo specific training in telephone triage and are supported, not only by a variety of health professionals, but by a computer decision support system (CDSS) called the ‘NHS Pathways’ [15, 59, 60]. A recent qualitative study suggested that call handlers will still utilise their own judgement and flexibility when using the ‘Pathways’ algorithm to interpret patient ‘risk’ [61]. This shift in responsibility shows that non-clinical staff can be trained to assess urgent calls in a safe and efficient way. Based on this, the veterinary profession should consider providing their client care teams with the same levels of training and support as their human medical counterparts. Yet, the findings of this study highlighted that client care teams have a diverse role within practice, from maintaining lines of communication and updating records to managing medication requests. As a consequence, the ever increasing complexity of their field may inadvertently prevent them from applying knowledge consistently and correctly. Additionally, even those with sufficient training or expertise can make occasional errors, a fact that has led to the adoption of ‘checklists’ in many industries, including Aviation and human medicine [62]. Therefore, it is imperative that standardised protocols or ‘checklists’ are used in conjunction with training instead of relying on one’s own judgement. The resources developed in this research study have been made freely available through the British Equine Veterinary Association website (https://portal.bevahosting.org.uk/Guidance-and-Resources/Practice-Managers/colic-resources), so they can be used in their current form, or adapted by veterinary practices to suit their individual requirements.

### Recommendations for future work

Colic is a common emergency condition encountered by veterinary practitioners, yet the results of this study suggest that the telephone triage of colic cases may currently be unstandardised within veterinary practice. Additionally, although client care teams were found to be most frequently managing telephone calls of this nature, this group were least confident in the telephone triage of colic with many reluctant to provide owners with basic advice. Though exploratory in nature, this study has highlighted a number of areas which require further investigation. Future studies should employ a quantitative approach in order to survey a larger sample of veterinary employees from both small and large animal establishments. These studies should focus on, prior training, current practice protocols and guidance on the telephone triage of veterinary emergencies in order to identify potential opportunities for further support, especially in relation to those with no formal clinical training. Given the predominant role client care teams perform within practice, industry stakeholders must ensure that these team members are fully supported. The role of client care teams must be acknowledged as more than just that of a ‘receptionist’, with training opportunities, including industry recognised qualifications, reflecting the skills level expected of those working within the socio-spatial environment of reception. Additionally, policy-makers within veterinary practice must work with their client care teams in order to identify barriers to knowledge transfer to ensure practice protocols are fully utilised. In line with our human medical counterparts, the development of a veterinary computer decision support system, supported by existing evidence and clinical expertise, could help to provide those triaging emergency telephone calls with additional guidance when interpreting case severity. Additionally, those with reservations about non-clinical staff providing advice must recognise that, as the first line of contact for a distressed owner, standardised non-clinical guidance could help to ensure the safety of both horse and owner in the absence of a qualified veterinary surgeon.

## Conclusion

This is the first study to report on the use of telephone triage for emergency conditions in equine practice. The study highlighted that many of the telephone calls are taken by client care teams, who may have varying experience and training. The information recorded and the advice given often varies, even within specific veterinary practices. The study used a participatory action research approach to develop and implement resources to support telephone triage and decision-making for colic in the horse. The resources developed have been made freely available to assist with the development of practice protocols and the development of further telephone triage systems and resources. However, further research is needed to ascertain the potential impact of these materials on the triage of emergency calls and case outcome. The study highlighted a need to recognise the importance of the client care team in recognising and triaging emergency cases, and provide consistent advice to owners. It identified a number of potential barriers to adopting standardised systems, including the existing recording systems within practices, but most significantly, perceptions around the role of the client care team in assessing and managing these calls. Industry and practice stakeholders must recognise the complex and challenging nature of working within the socio-spatial environment, and ensure that training opportunities and practice protocols reflect the perceived role of client care teams.

## Supporting information

S1 File Development and final design of an online survey to evaluate existing approaches and opinions of telephone triage of colic by members of veterinary practice teams. (DOCX)

S2 File Profiles of veterinary practices participating in an action research study developing and implementing telephone triage resources for colic in the horse. (DOCX)

S3 File Questionnaire provided to client care team members of four UK veterinary practices to evaluate personal confidence in taking telephone calls from owners of horses showing signs of colic before and after implementation of a resource to support decision-making. (DOCX)

S4 File Questionnaire provided to client care team members of four UK veterinary practices to evaluate feedback on resources to support decision-making during telephone triage of colic. (DOCX)

S5 File Verbal questionnaire provided to client care team members of four UK veterinary practices to evaluate approaches to telephone triage of horses with colic and opinions and implementation of new resources to support decision-making. (DOCX)

S6 File Personal reflexivity statement for a participatory action research study on telephone triage of colic in the horse. (DOCX)

S7 File Additional methods of recording information used by client care team members of four UK veterinary practices during telephone triage of horses with colic. (DOCX)

S8 File Clinical signs that veterinary practice staff associate with signs of colic from an online survey of UK veterinary practices approaches to telephone triage of colic in the horse. (DOCX)

S9 File Advice given by veterinary practice staff from an online survey of UK veterinary practices approaches to telephone triage of colic in the horse. (DOCX)
