# Supplementary material for: Mixed methods investigation of the use of telephone triage within UK veterinary practices for horses with abdominal pain: A Participatory action research study
Source: PLoS One. 2020 Sep 23;15(9):e0238874. doi: 10.1371/journal.pone.0238874 (PMC7510986; doi:10.1371/journal.pone.0238874)

A study overview, including a definition of the term ‘colic’ and research aims, was provided at the start of the survey and followed by a compulsory consent page. The initial survey design was comprised of three sections (Table 3-1) with logic applied at the start of section one to ensure those participating worked in practices which treated horses on a regular basis. Those who did not meet this criterion were redirected to a ‘thank you’ page and excluded from the remainder of the survey. An explanatory paragraph was included to introduce each new section.

**Table 3‑1**: Question type and topics including within the pilot version of an online survey investigating the telephone triage of colic within UK veterinary practices.

| **Section** | **Number of questions** | **Question Types** | **Reason for Inclusion** |
| --- | --- | --- | --- |
| ***Equine work*** | 1 | - 1 closed question with pre-defined answers | To ensure only those working within an establishment that regularly treats equids could participate |
| ***Section 1: ‘You and Your Practice’*** | 5 | - 2 closed questions with pre-defined answers - 3 open questions requiring free-text answers | Collect demographic information on the participant and their practice |
| ***Section 2: ‘Gathering Information’*** | 15 | - 8 numerical ratings (sliding scale) - 1 ranking of importance - 3 closed questions with pre-defined answers - 1 rating of confidence - 1 rating of difficulty - 1 optional comments box requiring a free-text answer | Ascertain how telephone calls are currently triaged by practices |
| ***Section 3: ‘REACT Now to Beat Colic’*** | 2 | - 1 closed questions with pre-defined answers | To establish awareness of the ‘REACT’ owner campaign |

Feedback resulted in the development and inclusion of an additional section focusing on the recognition and management of critical cases, including the use of four clinical vignettes. This included an open question exploring participant’s knowledge of potential indicators of critical cases (‘critical’ cases were defined as those requiring euthanasia or urgent medical / surgical intervention). In-depth clinical vignettes were used to represent two mild/medical types of colic and two describing critical/severe cases. Scenarios two and three specifically mentioned the term ‘colic’ within the text therefore, only scenarios one and four were accompanied by the closed-question *‘How likely do you think it is that this horse has colic?’* All scenarios were followed by the closed-question *‘How would you triage this call in terms of priority for a vet's visit?’* and two open questions which aimed to establish what information would be obtained during the telephone call and what advice would be given to the owner of the horse.


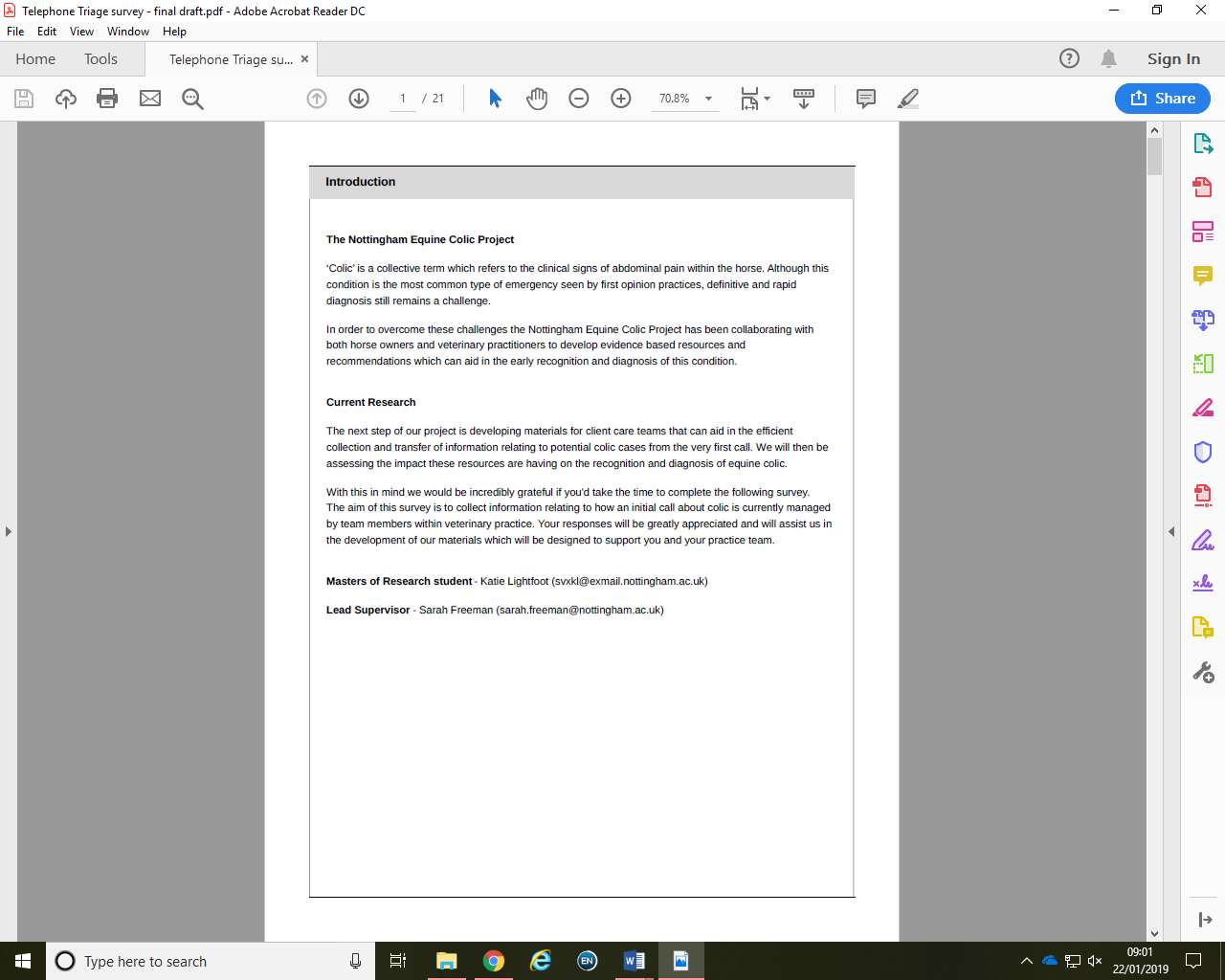

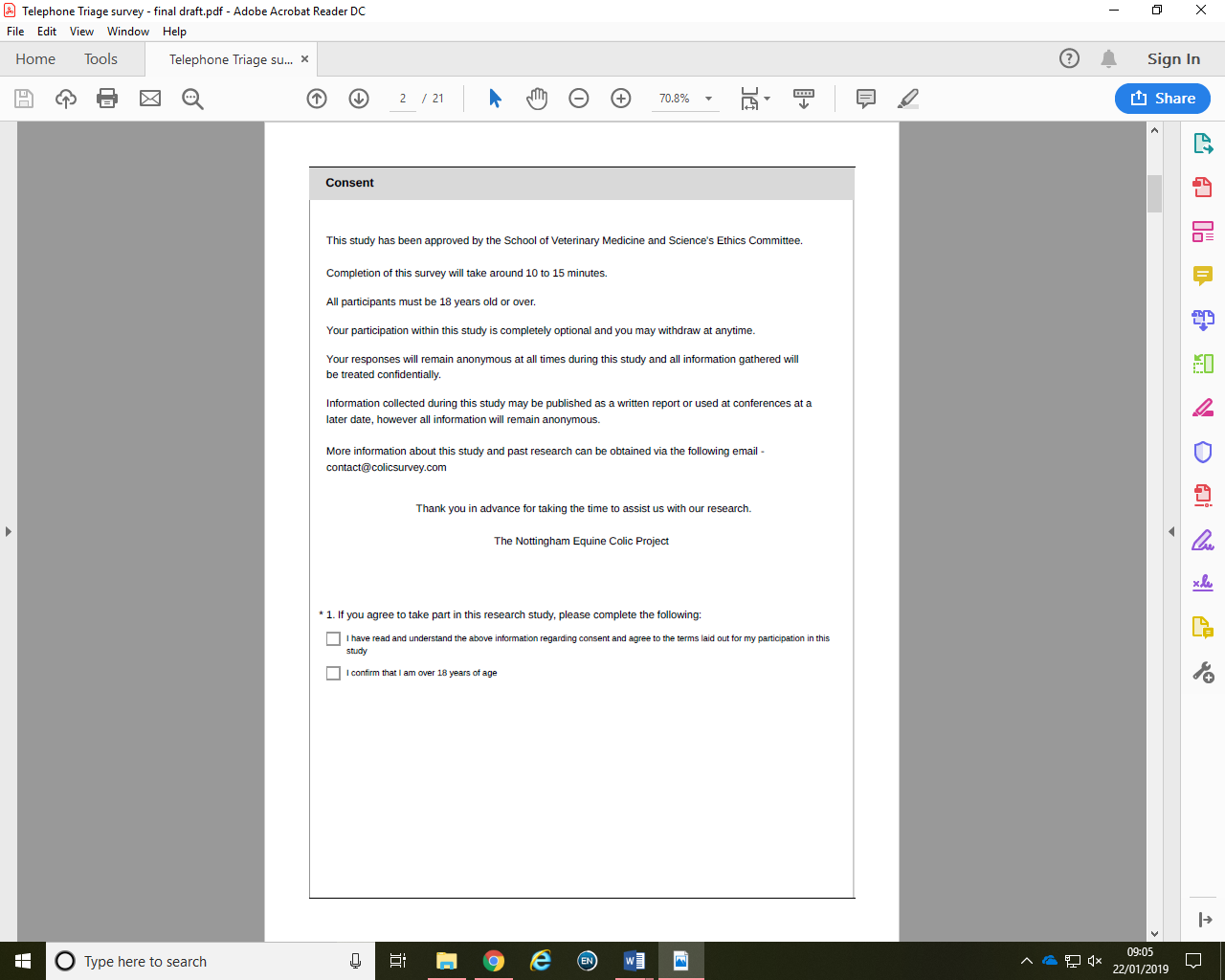


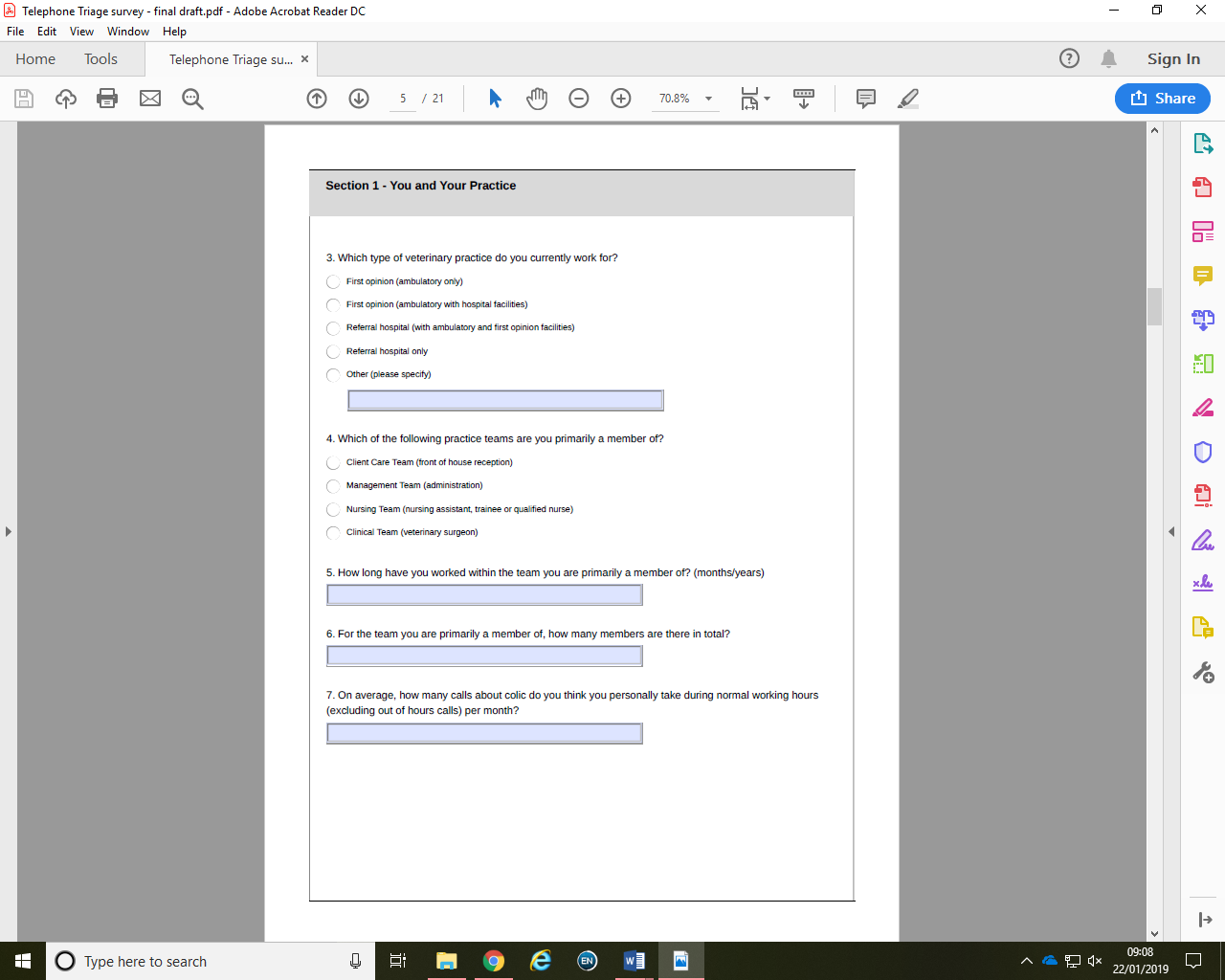

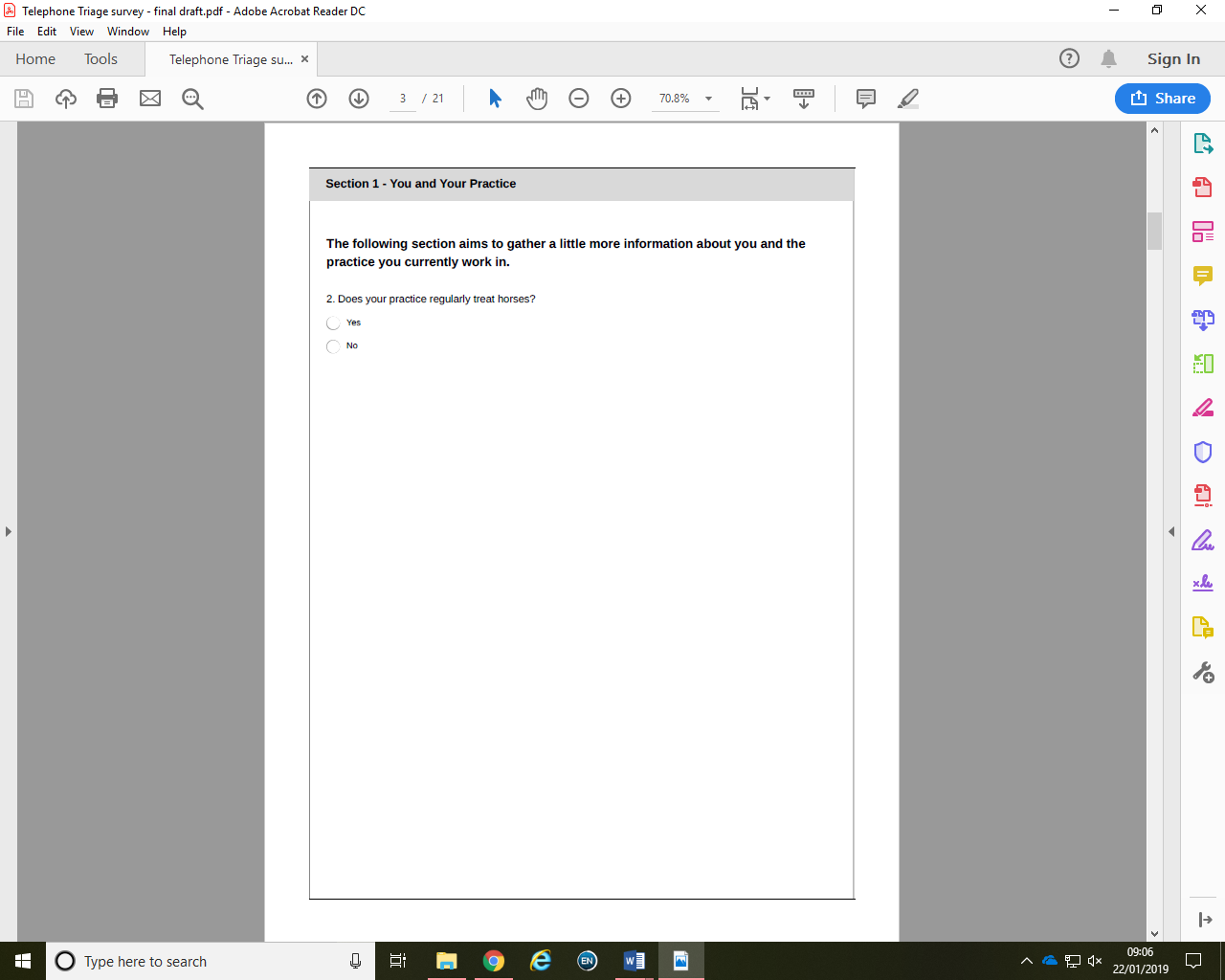


**
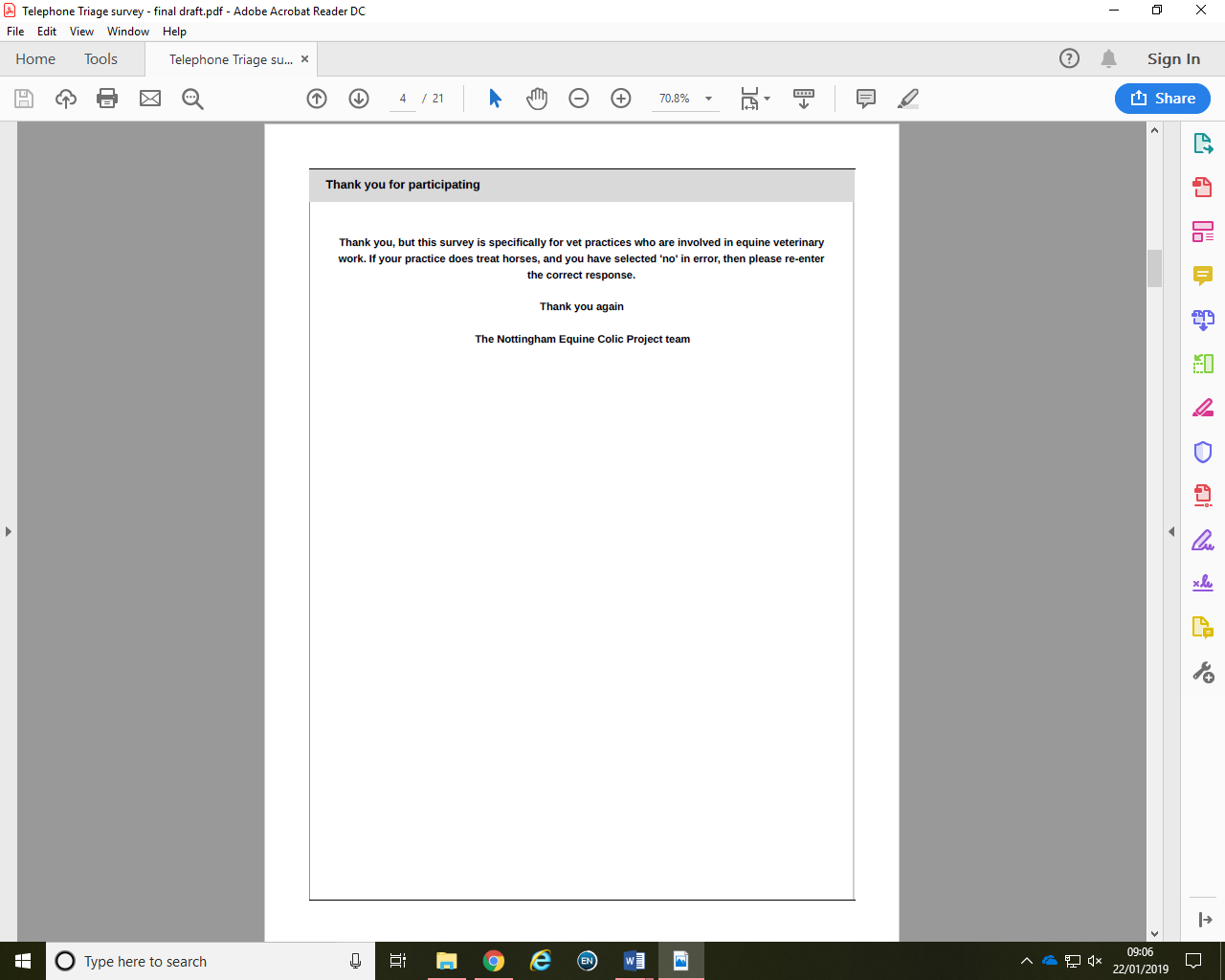
* Page displayed if answer to Q2 = No ***


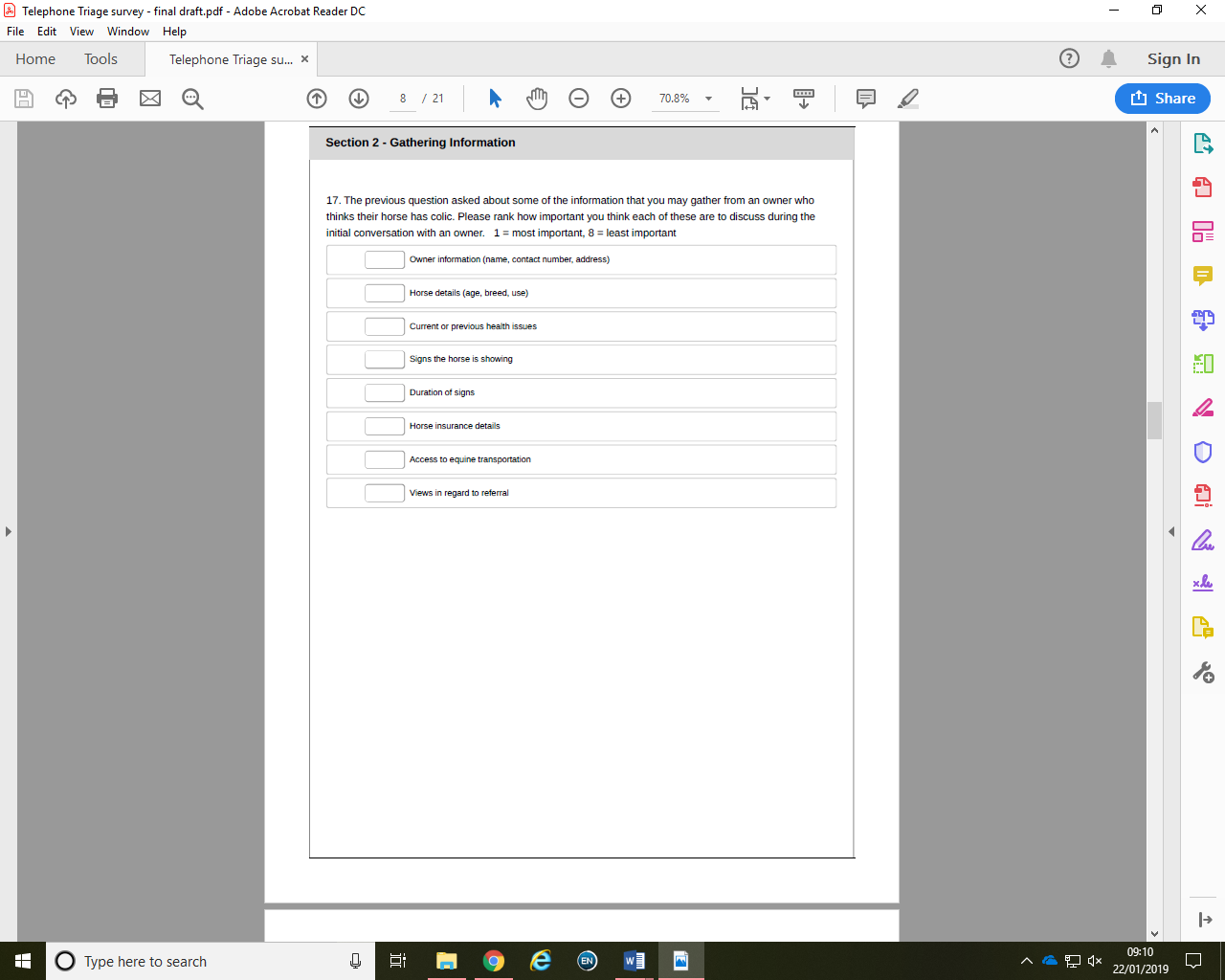

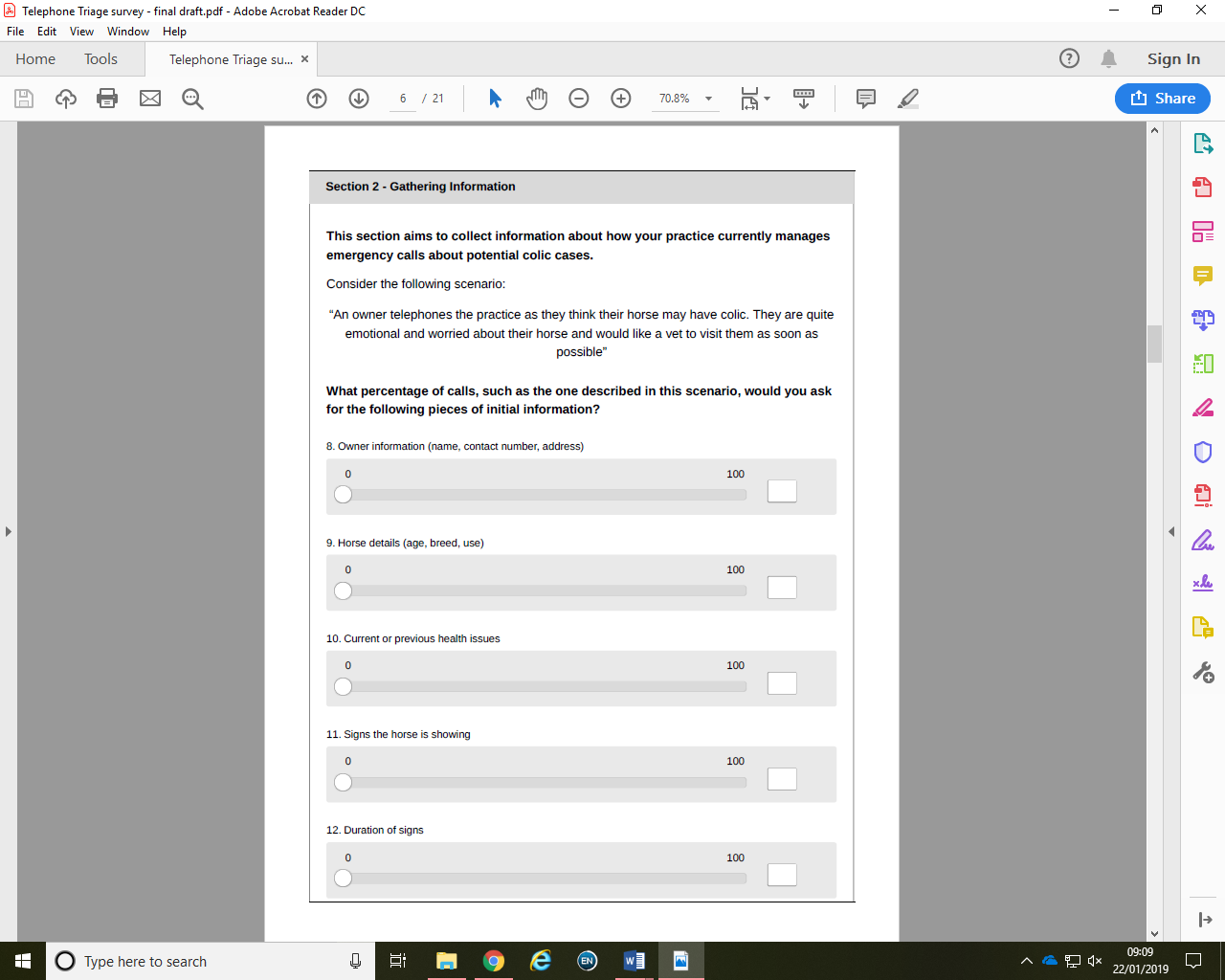

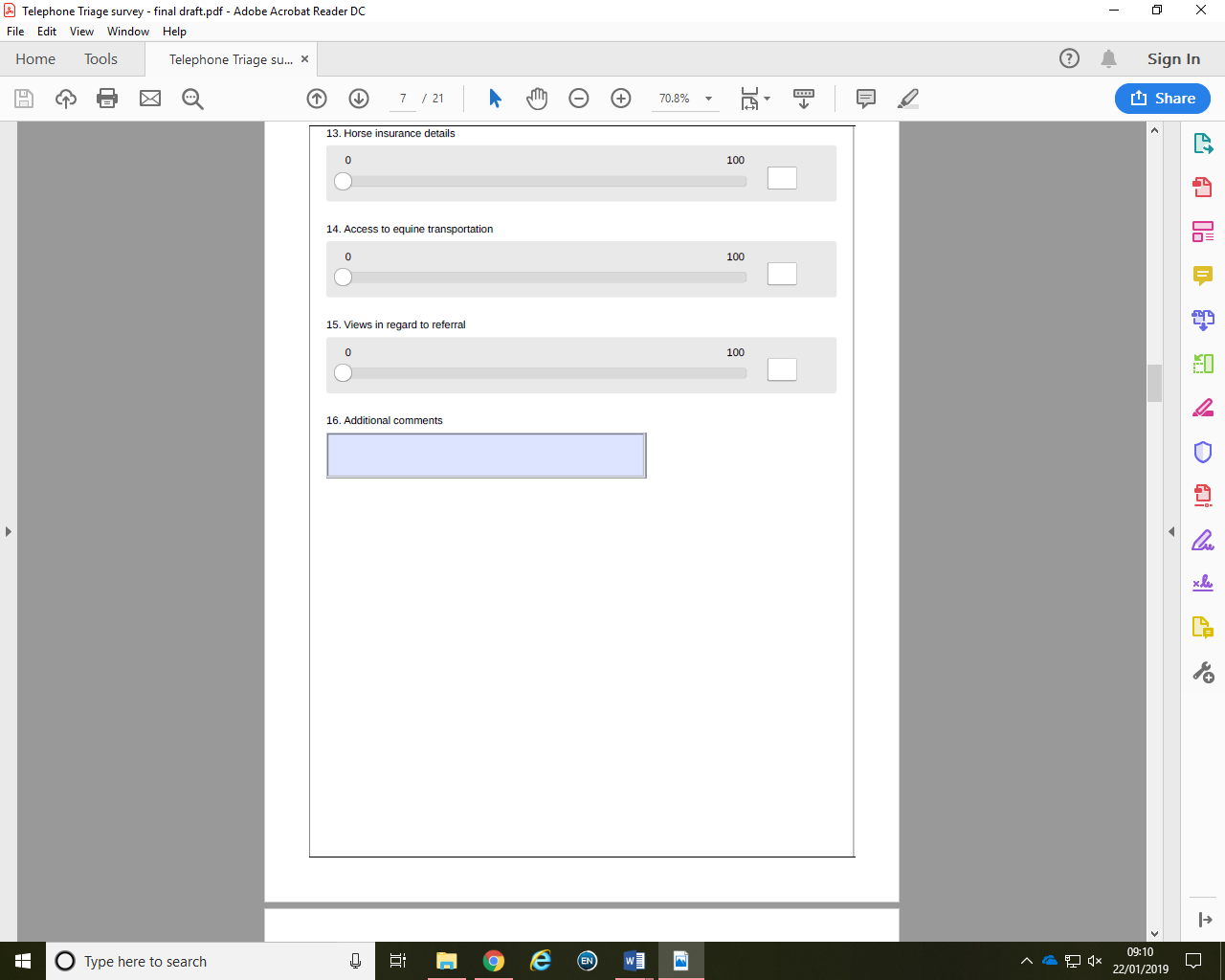


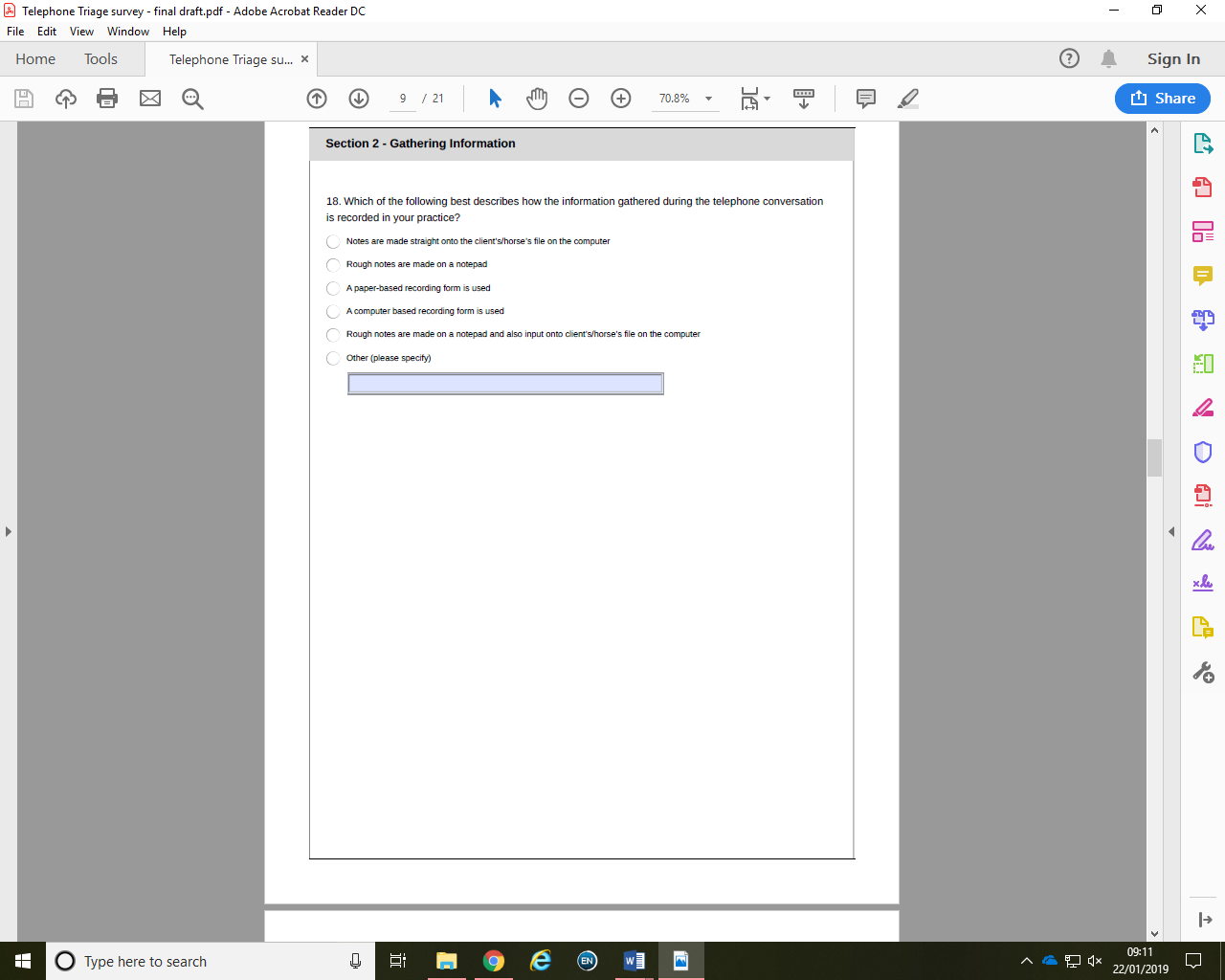

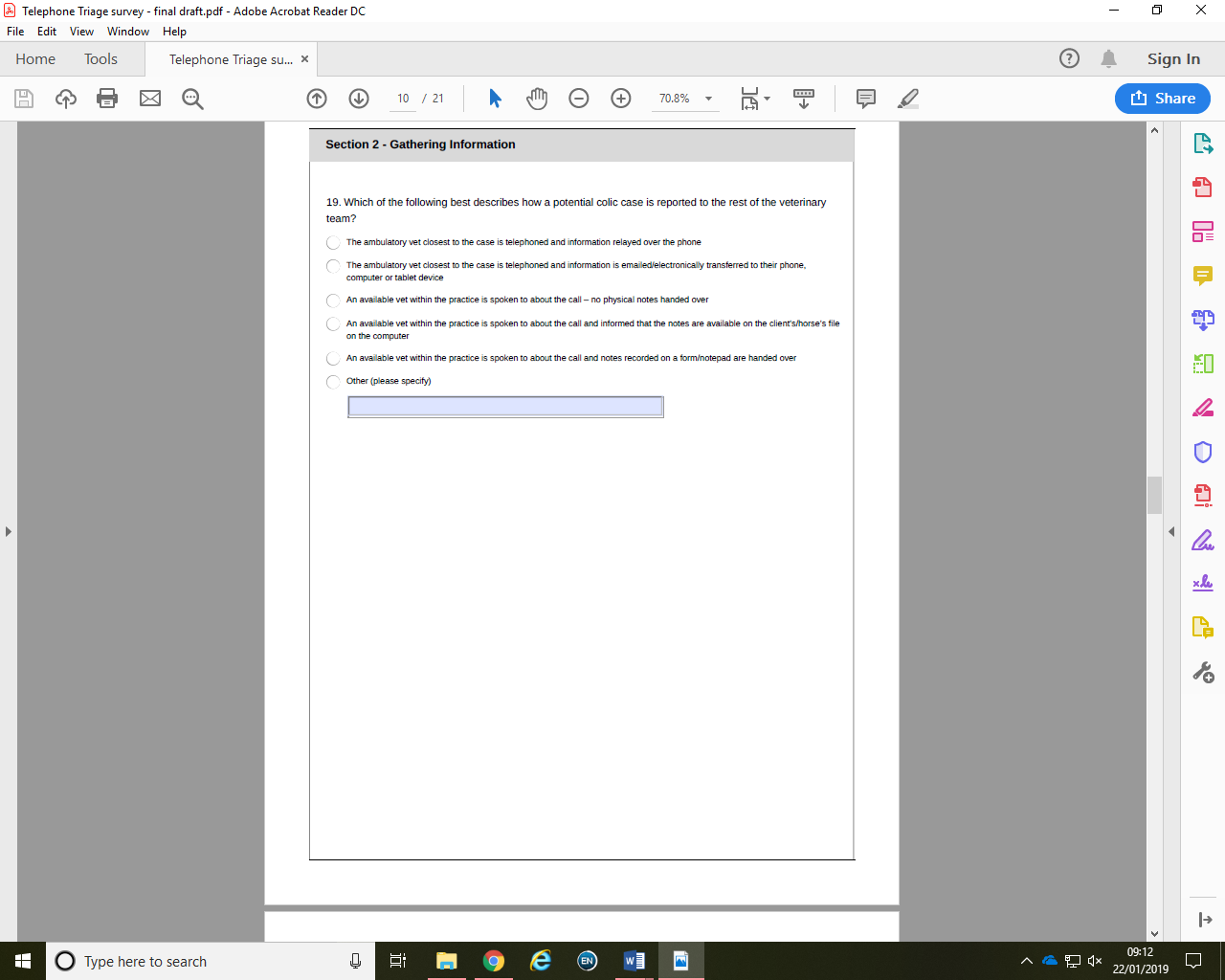

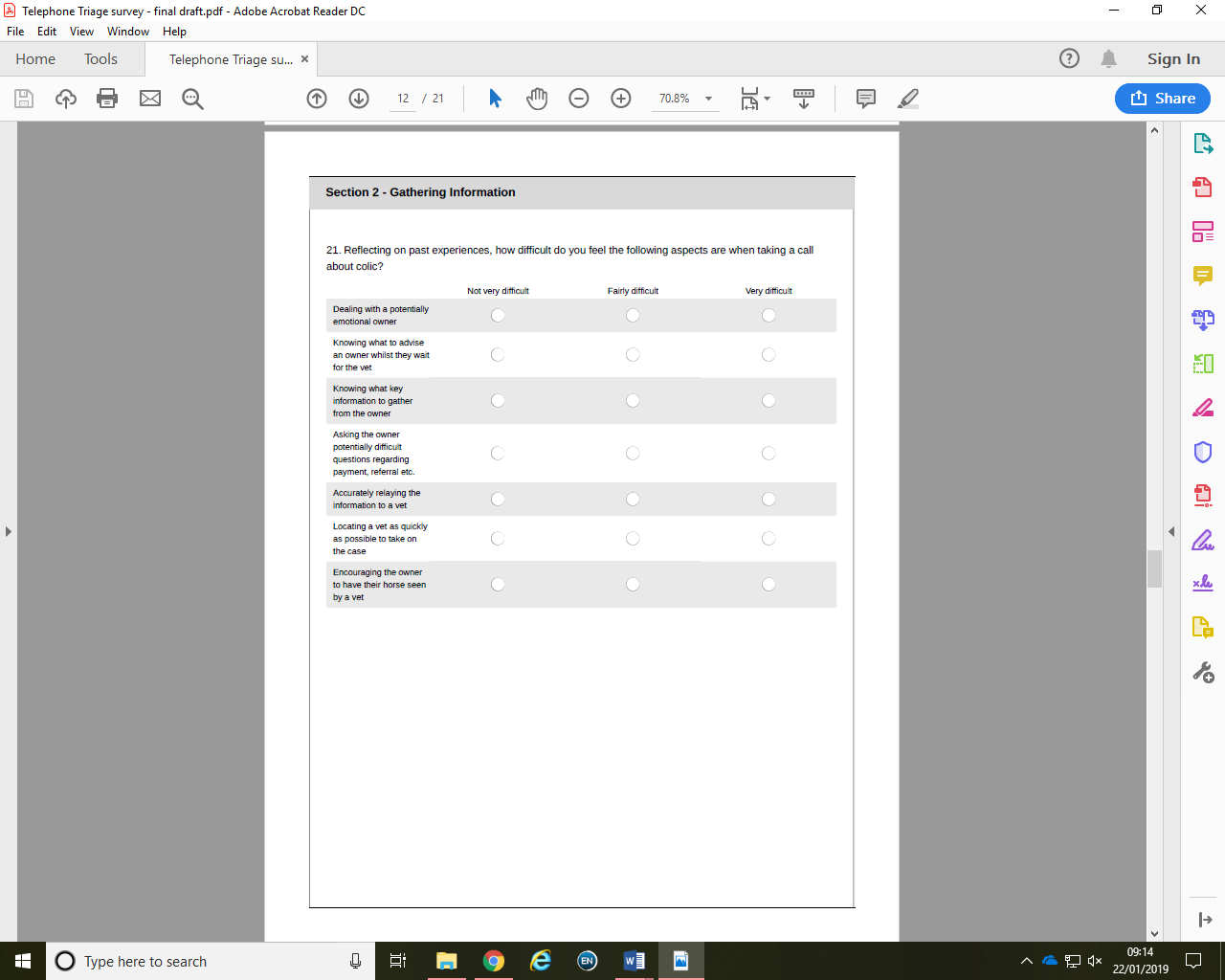

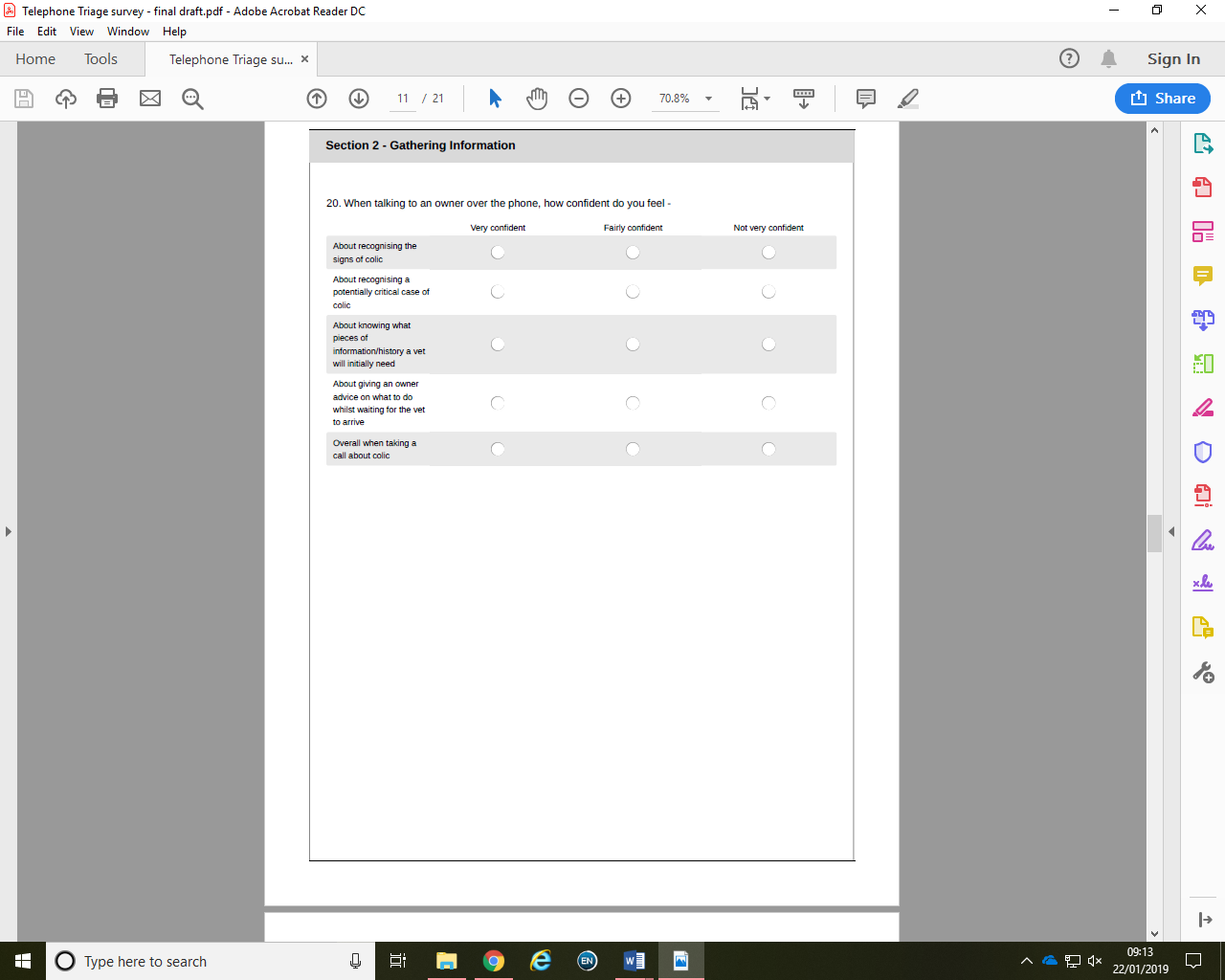


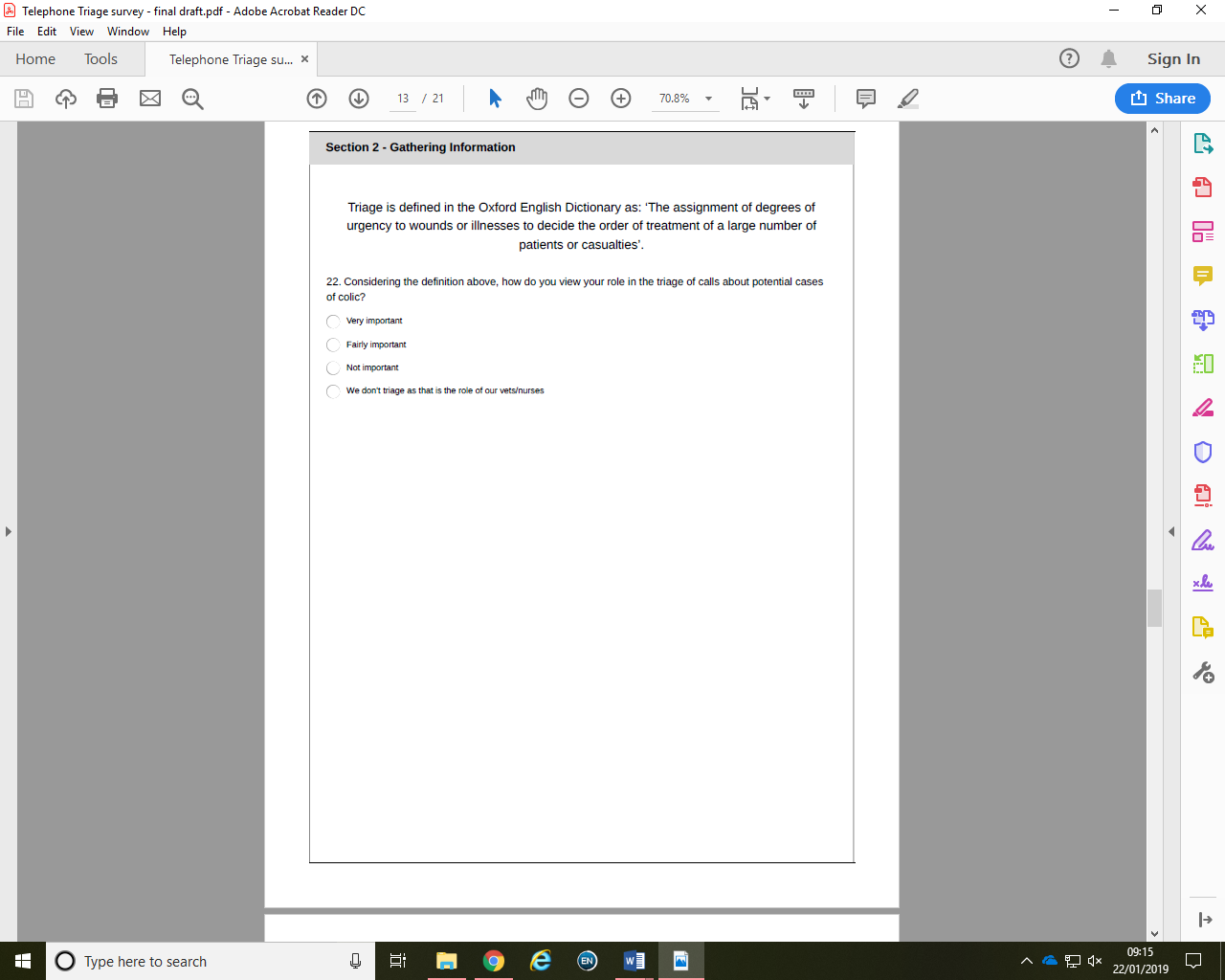

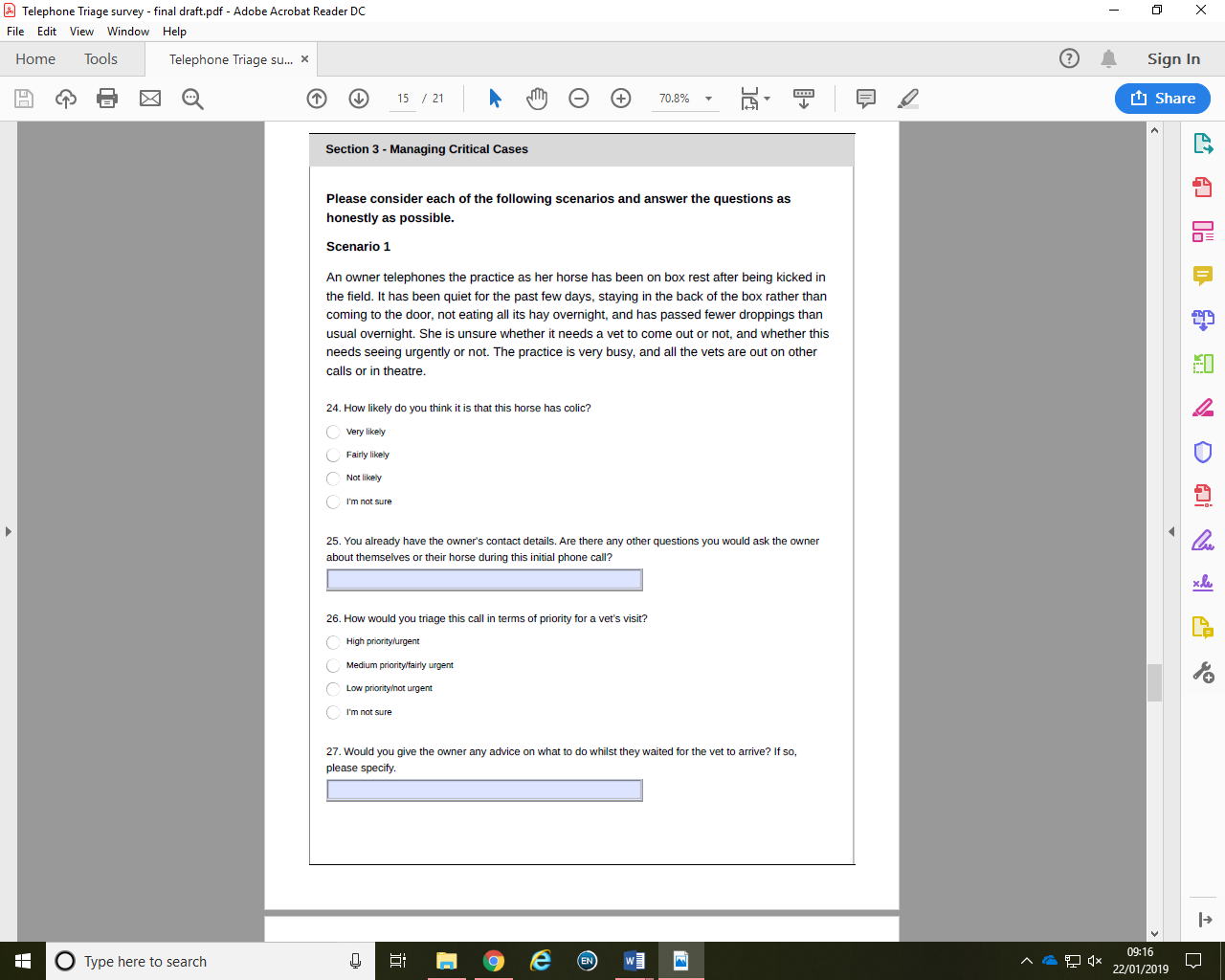

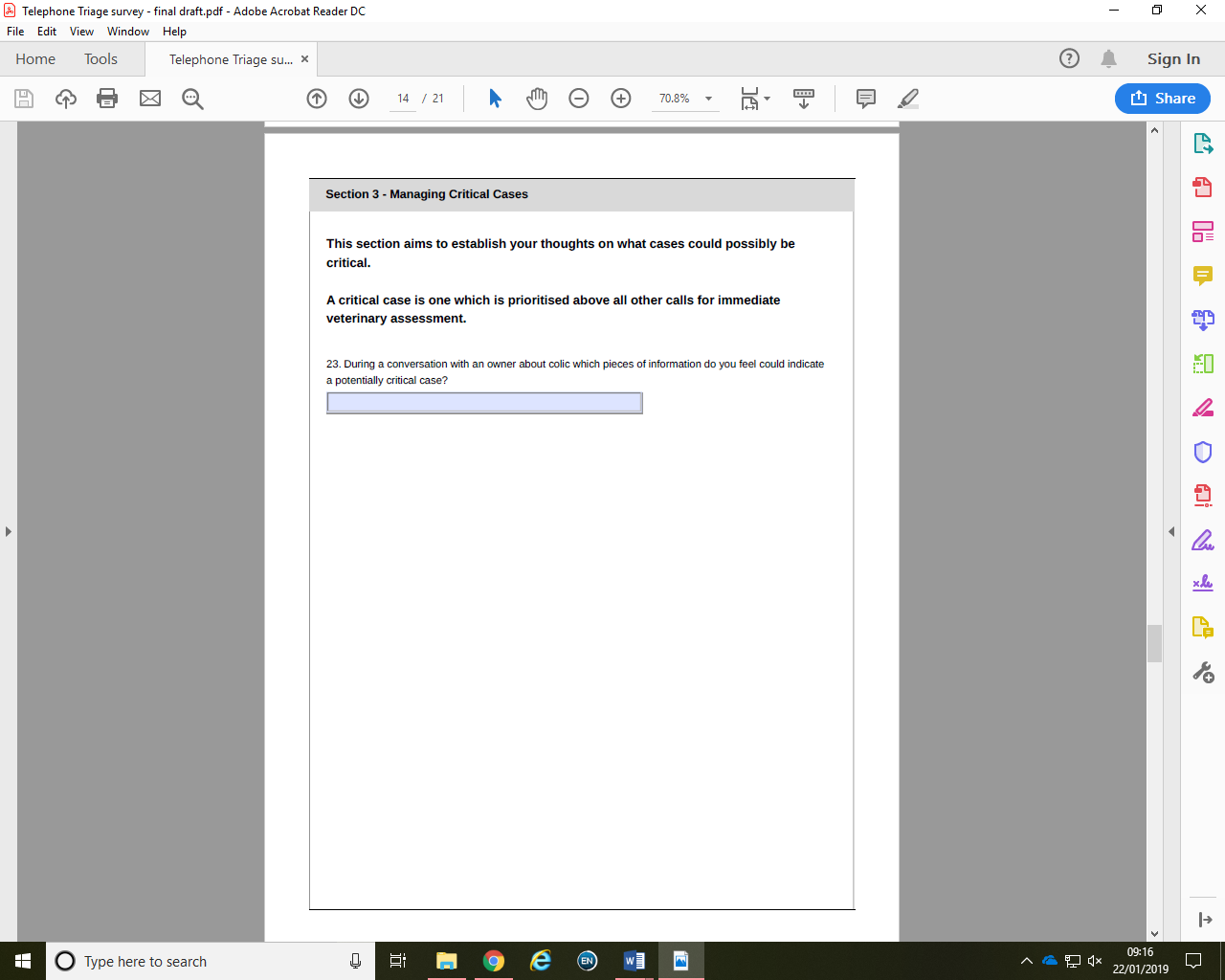


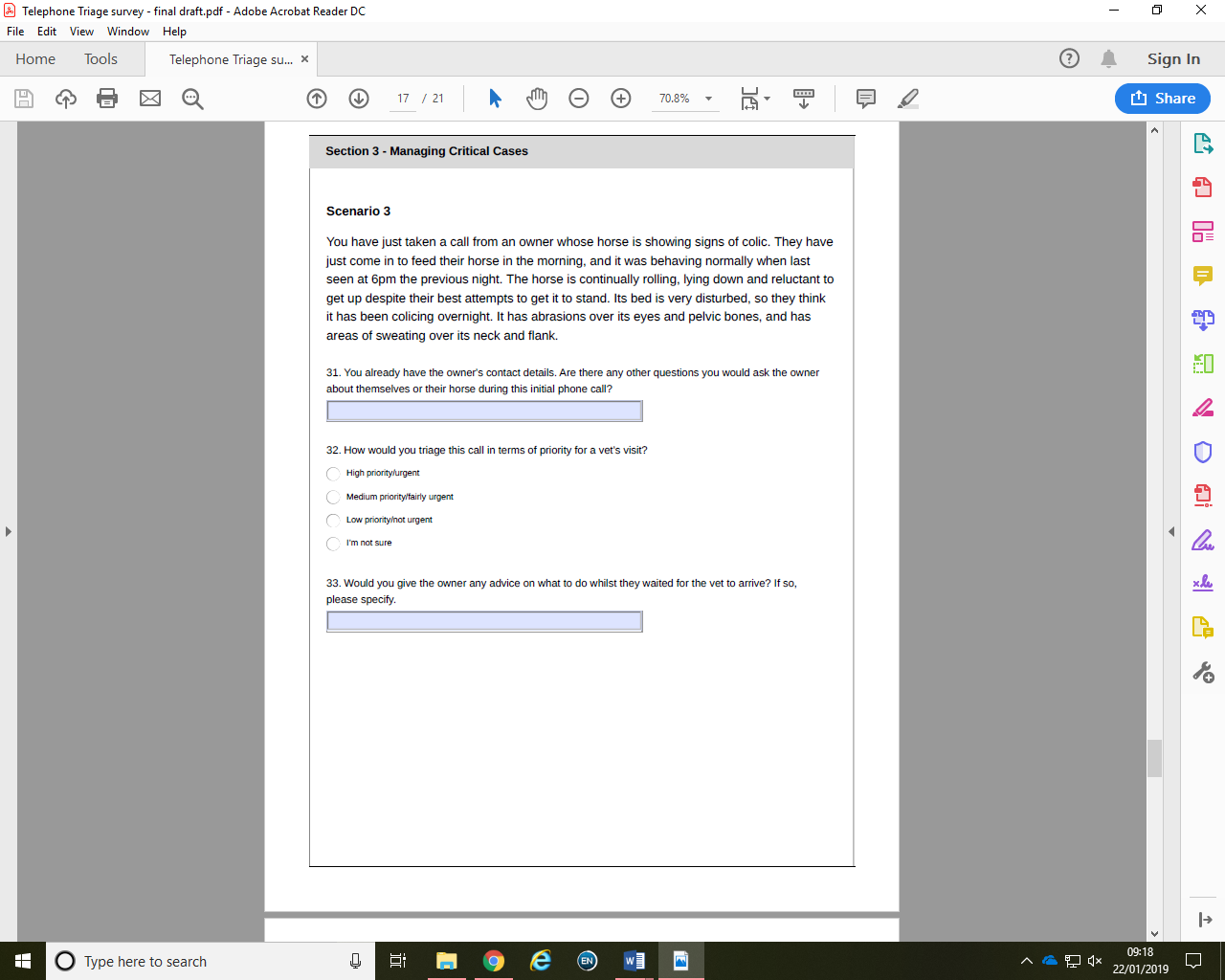

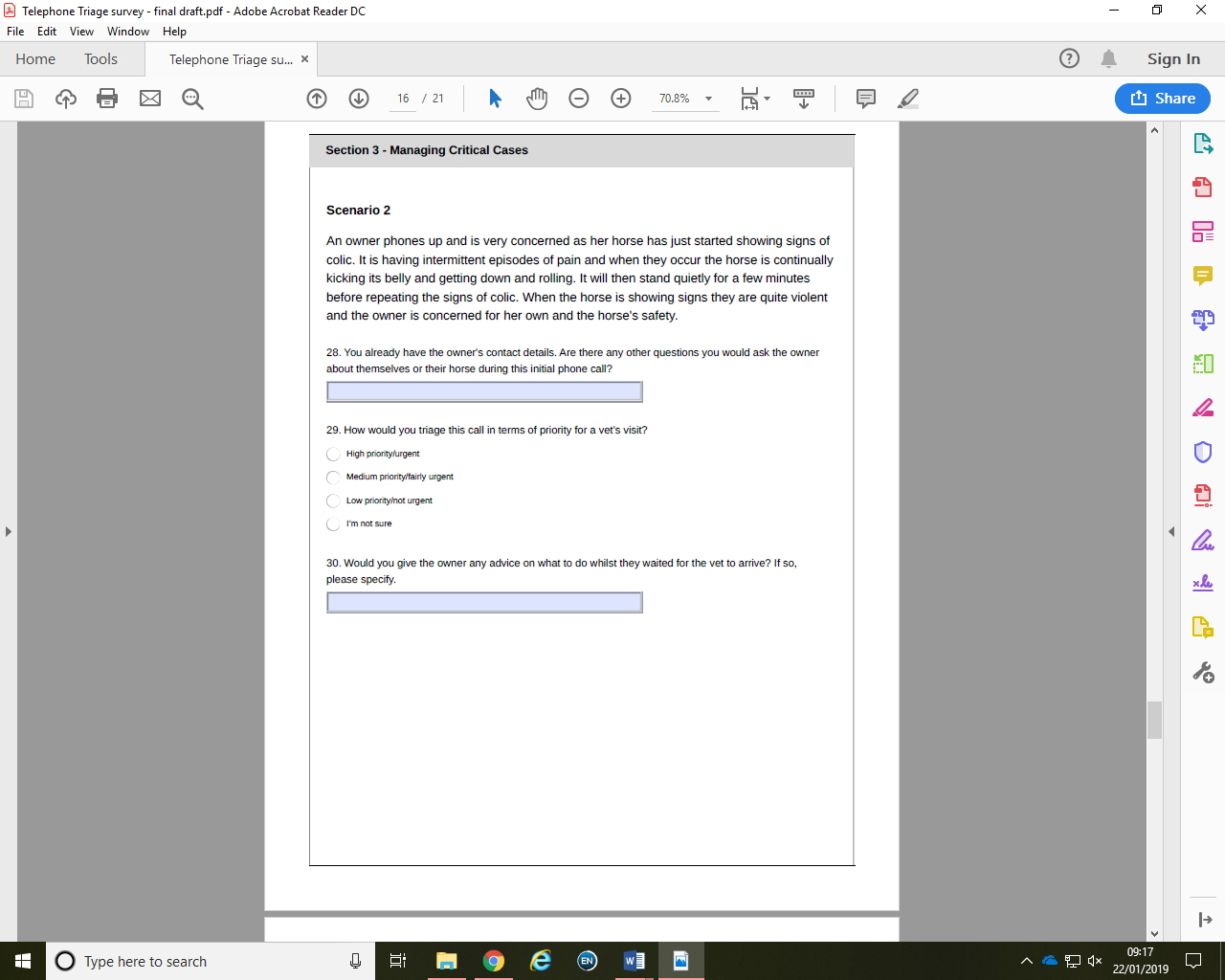


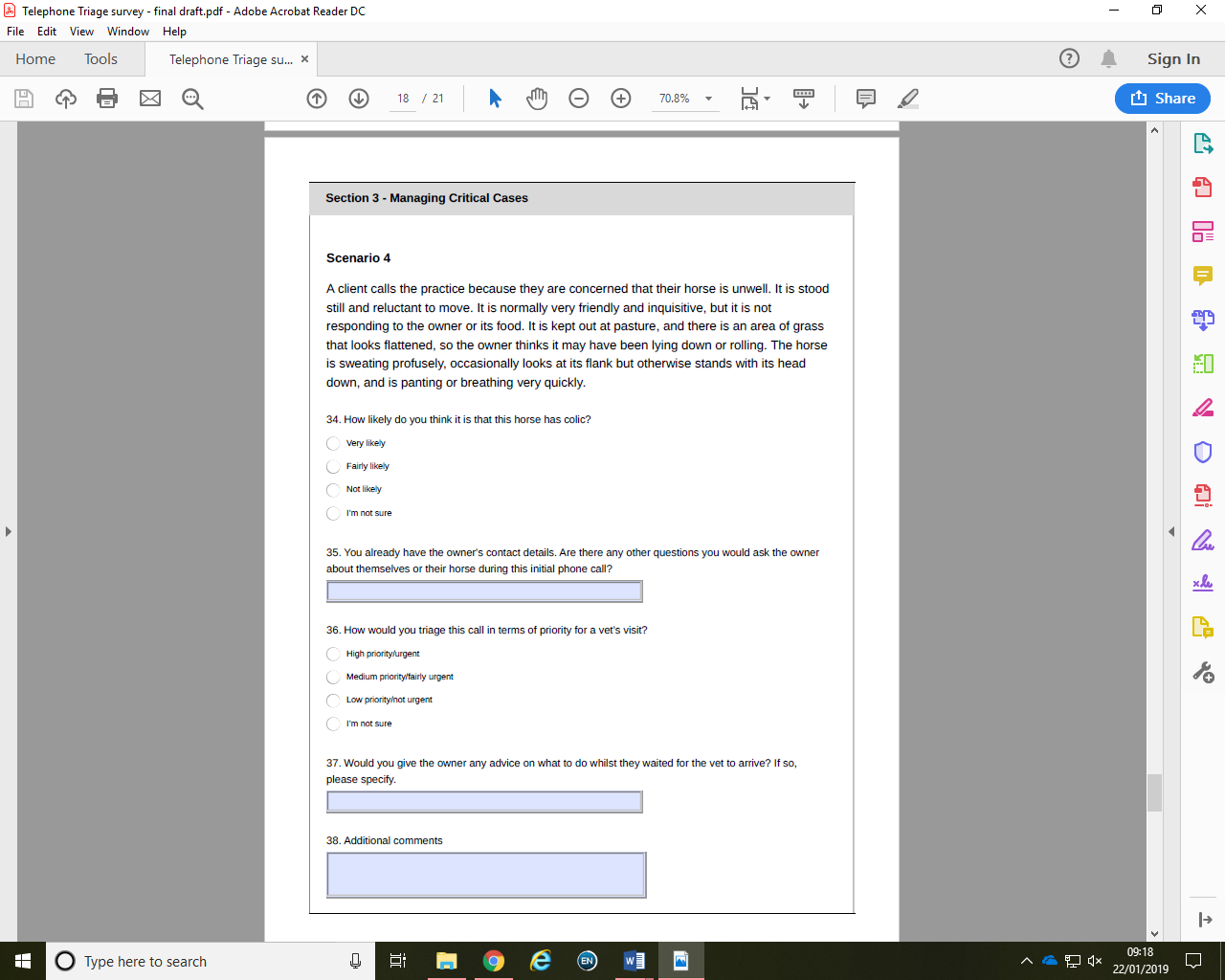

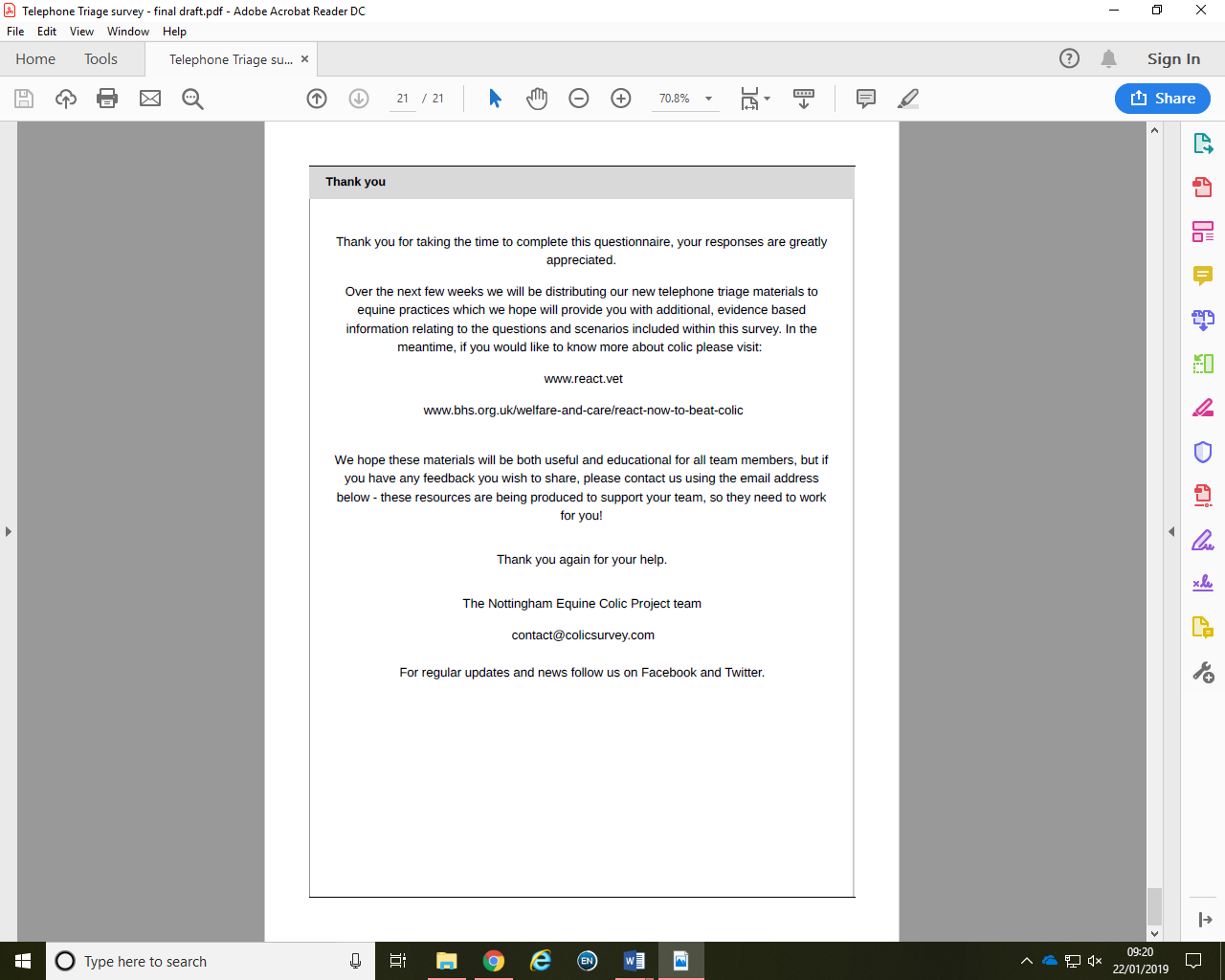

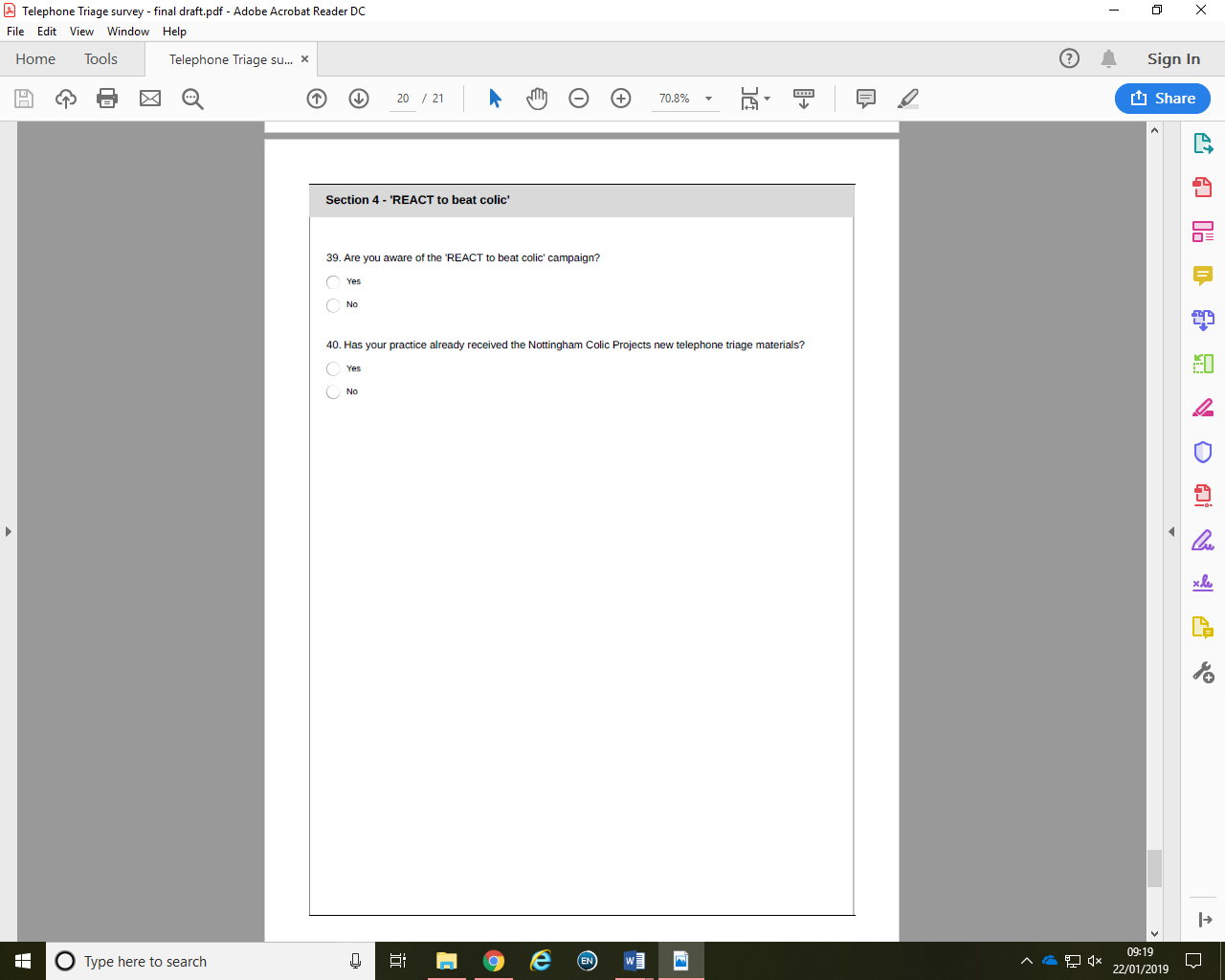

Supplement: S1 File — (DOCX) [file pone.0238874.s001.docx]
