# Supplementary material for: Mixed methods investigation of the use of telephone triage within UK veterinary practices for horses with abdominal pain: A Participatory action research study
Source: PLoS One. 2020 Sep 23;15(9):e0238874. doi: 10.1371/journal.pone.0238874 (PMC7510986; doi:10.1371/journal.pone.0238874)
Supplement: S2 File — (DOCX) [file pone.0238874.s002.docx]

| **Method of recording information whilst taking a telephone call** | **Number of participants**  **(n = 116)** |
| --- | --- |
| Notes are made straight onto the client’s/horse’s file on the computer | **n = 14** |
| Rough notes are made on a notepad | **n = 30** |
| A paper-based recording form is used | **n = 6** |
| A computer based recording form is used | **n = 8** |
| Rough notes are made on a notepad and also input onto client’s/horse’s file on the computer | **n = 49** |
| Other (please specify):  Paper/Practice Diary  Communicated in person/in writing and stored under PMS on client file  Vet would take these details in person when attending the call  Vet is verbally informed asap  The clients are well known so details are just checked  Computer diary | **n = 9**  (n = 3)  (n = 1)  (n = 1)  (n = 2)  (n = 1)  (n = 1) |
