# Supplementary material for: Mixed methods investigation of the use of telephone triage within UK veterinary practices for horses with abdominal pain: A Participatory action research study
Source: PLoS One. 2020 Sep 23;15(9):e0238874. doi: 10.1371/journal.pone.0238874 (PMC7510986; doi:10.1371/journal.pone.0238874)
Supplement: S3 File — (DOCX) [file pone.0238874.s003.docx]

The target population for this study were members of client care teams currently working within clinic-based or hospital practices, which provided first opinion services for equids on a daily basis. A convenience sample of four client care teams working within East Midlands veterinary practices were recruited to participate within this study. Practice profiles are as follows:

***Practice 1:*** An RCVS accredited hospital practice providing services for both companion animals and equids. This practice has a dedicated equine hospital and clinical team, which is separate to companion animal facilities. Equine reception is located within the small animal building in a dedicated office, where equine clinical team members can be reached via an internal telephone system. There are a total of eight client care team members who cover both small animal and equine reception on a rotational basis. First opinion and referral cases of colic are regularly seen, with intensive medical and surgical treatment performed.

***Practice 2:*** An RCVS accredited clinic-based practice providing services for both farm animals and equids. This practice has dedicated equine facilities and clinical personnel, separate to those utilised for farm animals. Reception is located at the front of the practice next to the clinical offices. Telephone queries from both farmers and equine clients are managed by the client care team, of which there are a total of six individual members at this practice. First opinion and simple medical colic cases are treated at this practice.

***Practice 3:*** An RCVS accredited hospital practice providing services for both companion animals and equids. This practice has a dedicated equine hospital and clinical team, which is separate to companion animal facilities. Equine reception is located upstairs within the main building, next to the clinical offices. There are two dedicated equine client care team members at this practice. First opinion and referral cases of colic are regularly seen, with intensive medical and surgical treatment performed.

***Practice 4:*** An RCVS accredited hospital practice providing services for both companion animals and equids. This practice has a dedicated equine hospital and clinical team, which is separate to companion animal facilities. Equine reception is situated in a dedicated area at the front of the practice close to clinical offices. There are a total of six dedicated equine client care team members at this practice. First opinion and referral cases of colic are regularly seen, with intensive medical and surgical treatment performed.
