# Supplementary material for: Mixed methods investigation of the use of telephone triage within UK veterinary practices for horses with abdominal pain: A Participatory action research study
Source: PLoS One. 2020 Sep 23;15(9):e0238874. doi: 10.1371/journal.pone.0238874 (PMC7510986; doi:10.1371/journal.pone.0238874)
Supplement: S4 File — (DOCX) [file pone.0238874.s004.docx]

**When talking to an owner over the phone, how confident do you feel:**

- **About recognising the signs of colic?**
- Very confident
- Fairly confident
- Not very confident
- **About recognising a potentially critical case of colic?**
- Very confident
- Fairly confident
- Not very confident
- **About knowing what pieces of information/history a vet will initially need?**
- Very confident
- Fairly confident
- Not very confident
- **About giving an owner advice on what to do whilst waiting for the vet to arrive?**
- Very confident
- Fairly confident
- Not very confident
- **Overall when taking a call about colic?**
- Very confident
- Fairly confident
- Not very confident
