# Supplementary material for: Mixed methods investigation of the use of telephone triage within UK veterinary practices for horses with abdominal pain: A Participatory action research study
Source: PLoS One. 2020 Sep 23;15(9):e0238874. doi: 10.1371/journal.pone.0238874 (PMC7510986; doi:10.1371/journal.pone.0238874)
Supplement: S5 File — (DOCX) [file pone.0238874.s005.docx]

| **Initial material feedback** | |
| --- | --- |
| 1. **Would you find the information within this pack useful**? Yes No | |
| 1. **Would you refer to this information when talking to owners/ taking calls?** Yes No | |
| 1. **What aspects of the information pack would you find useful?** | |
| 1. **What aspects of the information pack would you not find useful?** | |
| 1. **Would you find the colic recording form useful when taking a call?** Yes No | |
| *If YES why –* | *If NO why –* |
| 1. **Would you find the triage flow chart useful when taking a call?** Yes No | |
| If YES why – | If NO why- |
| ***Additional Comments:*** | |
