# Supplementary material for: Mixed methods investigation of the use of telephone triage within UK veterinary practices for horses with abdominal pain: A Participatory action research study
Source: PLoS One. 2020 Sep 23;15(9):e0238874. doi: 10.1371/journal.pone.0238874 (PMC7510986; doi:10.1371/journal.pone.0238874)
Supplement: S6 File — (DOCX) [file pone.0238874.s006.docx]

| **Question** | **Question Type** | **Reason For Inclusion** |
| --- | --- | --- |
| 1. What signs do you think relate to a potentially critical case of colic? | Open | Establish current methods of telephone triage and confidence relating to this topic. |
| 1. When talking to an owner over the phone, how confident, as a team, do you feel about –   Recognising signs of colic  Recognising critical cases  Knowing what information a vet will need  About giving an owner advice  Overall when taking a call about colic  (Pre-defined answers: Very confident, Fairly confident, Not very confident) | Rating of confidence |  |
| 1. Which three pieces of information do you feel are most important during an initial call about colic? | Open |  |
| 1. How do you currently record the information an owner gives you during a telephone conversation? | Open |  |
| 1. Has the information provided within the telephone triage pack had any impact upon the way you manage a potential call of equine colic? | Closed with pre-defined answers  Yes/No | Establish impact and use of telephone triage materials within practice  Establish impact and use of telephone triage materials within practice (cont.) |
| If **Yes** to Q5- Which aspects of the telephone triage pack have you found most useful?  If **No** to Q5 - Why do you think the materials haven’t had an impact? | Open |  |
| 1. Have you used the colic recording form in any way during a call about equine colic? | Closed with pre-defined answers  Yes/No |  |
| If **Yes** to Q6 - How have you been using the form?  If **No** to Q6 - Why do you think the form has not been used? | Open |  |
| 1. Have you used the triage flow chart in any way during a call about equine colic? | Closed with pre-defined answers  Yes/No |  |
| If **Yes** to Q7 – How has the triage flow chart been used?  If **No** to Q7 - Why do you think the triage flow chart has not been used? | Open |  |
| 1. Thinking about all of the information included within the telephone triage materials, is there any aspect or topic NOT covered that you would like more information on? | Closed with pre-defined answers  Yes/No |  |
| 1. Since being aware of the REACT owner campaign, have you noticed a difference in the way that owners report potential cases of colic? | Closed with pre-defined answers  Yes/No | Establish awareness of ‘REACT’ campaign and impact upon knowledge and approach |
| 1. Thinking about your own approach to potential calls about colic, has knowledge of the REACT owner campaign had any impact upon the way you now manage these calls? | Closed with pre-defined answers  Yes/No |  |
