# Supplementary material for: Mixed methods investigation of the use of telephone triage within UK veterinary practices for horses with abdominal pain: A Participatory action research study
Source: PLoS One. 2020 Sep 23;15(9):e0238874. doi: 10.1371/journal.pone.0238874 (PMC7510986; doi:10.1371/journal.pone.0238874)
Supplement: S7 File — (DOCX) [file pone.0238874.s007.docx]

**Personal Reflexivity Statement**

I am a white, female post-graduate student studying at the University of Nottingham’s School of Veterinary Medicine and Science. I grew up in the East Midlands and attended state school until the age of 16. After gaining a Diploma in Animal Management, I worked as an apprentice in the horse racing industry before being employed in customer services.

I entered the veterinary industry in my early twenties, with my first role being that of a Veterinary Receptionist in a small animal practice located in the East Midlands. I relished working in the fast paced environment of first-opinion practice and developed a keen interest in veterinary nursing and client education. Although I thoroughly enjoyed my time at this practice, my passion for horses ultimately led me to complete an undergraduate degree in Equine Sports Science. Alongside my studies, I worked part time as an equine nursing assistant in a first-opinion veterinary practice, as well as a curriculum support advisor at a local agricultural college.

During my time as a nursing assistant I witnessed many horse owners faced with the decision of caring for a severely ill or injured horse, with colic having the potential to be particularly distressing for all involved. These experiences, though difficult at times, provided me with an important insight into the horse-owner relationship, as well as a fundamental understanding of emergency veterinary care.

As a result, I undertook this PhD project to gain a better understanding of colic and the challenge this emergency condition presents situations. It is plausible to suggest that my experience in veterinary practice may have had some bearing on my interpretation of participant responses. However, I also have personal experience of caring for horses on a day to day basis. Additionally, although not a horse owner, I do care for animals who I adore. Having made numerous trips to veterinary establishments, both on a routine and emergency basis, I have experienced making difficult decisions as an animal owner. This, I believe, gives me the ability to not only understand the need for urgency in the event of a veterinary emergency, but to truly empathise with both veterinary teams and horse owners.

Before participating in interviews, participants were provided with an information sheet which briefly outlined my employment history and equine experience. Whilst this may have biased the answers they gave, I believe that this was minimised as much as was possible through the personalisation of interview schedules and maintaining focus on participants own experiences.
