# Supplementary material for: Mixed methods investigation of the use of telephone triage within UK veterinary practices for horses with abdominal pain: A Participatory action research study
Source: PLoS One. 2020 Sep 23;15(9):e0238874. doi: 10.1371/journal.pone.0238874 (PMC7510986; doi:10.1371/journal.pone.0238874)
Supplement: S8 File — (DOCX) [file pone.0238874.s008.docx]

| **Indicator of 'critical' colic** | **Percentage of participants reporting sign** |
| --- | --- |
| Duration of signs | 38.5% (42/109) |
| Severity of signs | 33.9% (37/109) |
| Rolling | 23.9% (26/109) |
| Sweating | 17.4% (19/109) |
| 'General' signs (unspecified) | 13.8% (15/109) |
| Violent signs (unspecified) | 11.0% (12/109) |
| Clinical signs (unspecified) | 10.1% (11/109) |
| Previous history and / or episodes of colic | 9.2% (10/109) |
| Recumbancy | 9.2% (10/109) |
| Reduced droppings | 6.4% (7/109) |
| Signs of discomfort | 6.4% (7/109) |
| Dull demeanour | 5.5% (6/109) |
| Increased heart rate | 5.5% (6/109) |
| Kicking at stomach | 4.6% (5/109) |
| Reluctance to stand | 4.6% (5/109) |
| Abnormal Mucous Membrane Colour | 3.7% (4/109) |
| Nasal discharge / Spontaneous reflux | 3.7% (4/109) |
| Abrasions | 2.8% (3/109) |
| Distressed behaviour | 2.8% (3/109) |
| Increased respiratory rate | 2.8% (3/109) |
| Abdominal distension | 1.8% (2/109) |
| Agitation | 1.8% (2/109) |
| Collapse | 1.8% (2/109) |
| Depressed | 1.8% (2/109) |
| Knowledge or concern level of owner | 1.8% (2/109) |
| Pawing the ground | 1.8% (2/109) |
| Shaking | 1.8% (2/109) |
| Attempts to regurgitate/vomit | 0.9% (1/109) |
| Inability to pass faeces/urinate | 0.9% (1/109) |
| Increased temperature | 0.9% (1/109) |
| Lethargy | 0.9% (1/109) |
| Practitioners treat all cases as critical | 0.9% (1/109) |
| Reduced appetite | 0.9% (1/109) |
| Reduced gut sounds | 0.9% (1/109) |
| Reluctance to move | 0.9% (1/109) |
| Vocalisation | 0.9% (1/109) |
| All cases treated as critical - not our role to decide | 5.5% (6/109) |
| Unsure | 0.9% (1/109) |
