# Supplementary material for: Mixed methods investigation of the use of telephone triage within UK veterinary practices for horses with abdominal pain: A Participatory action research study
Source: PLoS One. 2020 Sep 23;15(9):e0238874. doi: 10.1371/journal.pone.0238874 (PMC7510986; doi:10.1371/journal.pone.0238874)
Supplement: S9 File — (DOCX) [file pone.0238874.s009.docx]

| **Advice Topic** | **Number of participants reporting advice topic for each scenario** | | | | **Total times advice topic reported** |
| --- | --- | --- | --- | --- | --- |
|  | **Scenario 1** | **Scenario 2** | **Scenario 3** | **Scenario 4** |  |
| Safety – both horse and owner | 11.7% (12/103) | 53.3% (57/107) | 47.3% (44/93) | 19.0% (16/84) | 129 |
| Exercise the horse | 31.1% (32/103) | 36.4% (39/107) | 16.1% (15/93) | 15.5% (13/84) | 99 |
| Remove any remaining feed | 29.1% (30/103) | 15.9% (17/107) | 7.5% (7/93) | 10.7% (9/84) | 63 |
| Monitor the horse | 24.3% (25/103) | 7.5% (8/107) | 9.7% (9/93) | 8.3% (7/84) | 49 |
| Not allowed to provide advice | 9.7% (10/103) | 9.3% (10/107) | 10.8% (10/93) | 11.9% (10/84) | 40 |
| Would not give advice for this scenario | 11.7% (12/103) | 2.8% (3/107) | 8.6% (8/93) | 13.1% (11/84) | 34 |
| Leave horse alone / in stable | 2.9% (3/103) | 2.8% (3/107) | 12.9% (12/93) | Not reported | 28 |
| Call practice if concerned | 20.4% (21/103) | 2.8% (3/107) | Not reported | 3.6% (3/84) | 27 |
| Consult practice vet and relay information | 3.9% (4/103) | 7.5% (8/107) | 5.4% (5/93) | 1.2% (1/84) | 18 |
| Prepare for possible referral / vets arrival | Not reported | 1.9% (2/107) | 8.6% (8/93) | 4.8% (4/84) | 14 |
| Bring horse into a stable | Not reported | Not reported | Not reported | 16.7% (14/84) | 14 |
| Comfort the horse / keep calm | 1.0% (1/103) | Not reported | 3.2% (3/93) | 6.0% (5/84) | 9 |
| Encourage the horse to drink | 5.8% (6/103) | Not reported | Not reported | 3.6% (3/84) | 9 |
| Do not walk the horse | Not reported | Not reported | Not reported | 16.7% (14/84) | 4 |
| Take rectal temperature | 1.9% (2/103) | Not reported | Not reported | 1.2% (1/84) | 3 |
| Do not give any medication | Not reported | 0.9% (1/017) | Not reported | Not reported | 3 |
| Rug / change rug | Not reported | Not reported | Not reported | 3.6% (3/84) | 3 |
| Offer the horse damp hay | 1.0% (1/103) | Not reported | Not reported | Not reported | 1 |
| Remove water | Not reported | Not reported | Not reported | 1.2% (1/84) | 1 |
